# Testosterone Affects Neural Gene Expression Differently in Male and Female Juncos: A Role for Hormones in Mediating Sexual Dimorphism and Conflict

**DOI:** 10.1371/journal.pone.0061784

**Published:** 2013-04-16

**Authors:** Mark P. Peterson, Kimberly A. Rosvall, Jeong-Hyeon Choi, Charles Ziegenfus, Haixu Tang, John K. Colbourne, Ellen D. Ketterson

**Affiliations:** 1 Department of Biology, Center for Integrative Study of Animal Behavior, Indiana University, Bloomington, Indiana, United States of America; 2 Center for Genomics and Bioinformatics, Indiana University, Bloomington, Indiana, United States of America; 3 Cancer Center, Department of Biostatistics, Augusta, Georgia, United States of America; 4 Department of Mathematics, James Madison University, Harrisonburg, Virginia, United States of America; 5 School of Biosciences, University of Birmingham, Birmingham, United Kingdom; CNRS, Université de Bourgogne, France

## Abstract

Despite sharing much of their genomes, males and females are often highly dimorphic, reflecting at least in part the resolution of sexual conflict in response to sexually antagonistic selection. Sexual dimorphism arises owing to sex differences in gene expression, and steroid hormones are often invoked as a proximate cause of sexual dimorphism. Experimental elevation of androgens can modify behavior, physiology, and gene expression, but knowledge of the role of hormones remains incomplete, including how the sexes differ in gene expression in response to hormones. We addressed these questions in a bird species with a long history of behavioral endocrinological and ecological study, the dark-eyed junco (*Junco hyemalis*), using a custom microarray. Focusing on two brain regions involved in sexually dimorphic behavior and regulation of hormone secretion, we identified 651 genes that differed in expression by sex in medial amygdala and 611 in hypothalamus. Additionally, we treated individuals of each sex with testosterone implants and identified many genes that may be related to previously identified phenotypic effects of testosterone treatment. Some of these genes relate to previously identified effects of testosterone-treatment and suggest that the multiple effects of testosterone may be mediated by modifying the expression of a small number of genes. Notably, testosterone-treatment tended to alter expression of different genes in each sex: only 4 of the 527 genes identified as significant in one sex or the other were significantly differentially expressed in both sexes. Hormonally regulated gene expression is a key mechanism underlying sexual dimorphism, and our study identifies specific genes that may mediate some of these processes.

## Introduction

Selection often favors different traits or trait values in males and females, giving rise to sexually antagonistic selection [1]–[3]. These sexually antagonistic patterns of selection have the potential to constrain evolution [4], resulting in less fit intermediate phenotypes [5]–[7], even in the face of strong selection [8]. As a consequence, genetic, developmental and physiological mechanisms that favor sex-specific phenotypes and sexual dimorphism are expected to be favored, thus reducing sexual conflict.

One way to achieve sexual dimorphism and to relieve sexual conflict is to regulate conflicting traits with signaling molecules that circulate at different levels in the two sexes [9]. Specifically, testosterone (T) is a steroid hormone that circulates at higher levels in males than females in many species and regulates a number of sexually dimorphic phenotypes including: sexual signals [10], aggression [11], breeding state [12], and courtship behavior [13]. In many temperate-zone songbirds, males sing during the breeding season and females do not, and the seasonal shift to singing behavior is mediated by T in males [14], [15]. In some species, females exposed to experimentally elevated T develop male-like neuroanatomy and can be induced to sing ([16]; reviewed in [17]), suggesting that adult sex differences in hormone levels give rise to some sexual dimorphisms.

Males and females share largely identical genomes, and sexually dimorphic behavior in many species is thought to arise from sexually dimorphic gene expression (reviewed in [18]). For example, whole brains in songbirds show marked sex differences in the expression of hundreds of genes [19], and differences in gene expression between the sexes in the brains of cichlid fish (*Astatotilapia burtoni*), outnumber those between two phenotypically divergent alternative male phenotypes [20]. Sex-biased gene expression has been related to sex-biased behaviors including sexual performance, aggression, and parental care [21], and steroid hormone levels, including T, are known to affect sexually dimorphic gene expression during development [21], [22] and adulthood [23].

Levels of T, however, appear to be correlated between males and females across species, which creates the potential for conflict over optimal circulating levels [24]–[26] given that traits that are beneficial in males may be detrimental in females [17]. Following this reasoning, selection might be expected to favor females with reduced capacity to respond to T (sensitivity to T) through one of many possible mechanisms [17], [27]. For example, aggression is influenced by (sensitive to) experimentally elevated T in female tree swallows (*Tachycineta bicolor*) [28], zebra finches (*Taeniopygia guttata*) [29] and red-winged blackbirds (*Agelaius phoeniceus*) [30], but is insensitive to T in female European robins (*Erithacus rubecula*) [31] and European starlings (*Sturnus vulgaris*) [32]. Such species differences in female sensitivity to T suggest that evolution can shield one sex from possible detrimental effects of selection on the other sex. The mechanisms for this shielding are still unknown, and changes in gene expression response to T may be important.

While past research has provided important insights into sexual dimorphism and the role of hormones in regulating phenotype, far less is known about the role of hormones in regulating the sex-specific gene expression that underlies these phenotypes. By bringing genomic tools to a system whose natural ecology is well known, greater understanding of the production and maintenance of sexual dimorphism should be possible.

We measured gene expression in two brain regions in males and females of a songbird, the dark-eyed junco (*Junco hyemalis*) using a species-specific microarray. We also measured the effect of exposure to experimentally elevated testosterone on gene expression by comparing experimental animals to controls of each sex. The junco is an avian system with mild sexual dimorphism [33], and its behavior, ecology, and physiology have been extensively studied over the past century [34]–[37]. Experimental and correlative studies of natural populations of the junco have linked hormonal variation to variation in phenotype [36], [38] and natural selection [39], [40] in the wild. The hormonal treatment used here has been utilized extensively in juncos [41]–[47] and other species [12], [16], [48]–[52] to induce behavioral and physiological changes that last several months [53], [54].

While many traits are related to T in both sexes of the junco, there are some traits for which females appear to be behaviorally or physiologically insensitive to T (reviewed in [17]). In both male and female juncos, higher T is related to higher aggression [43], [55], lower body-mass [45], [56], and lower immune function [43], [44]. In contrast, several phenotypes that respond to experimentally elevated T in males do not respond in females, including nestling provisioning [41], [45], [46], [57], which declines only in males, and home range size [58], [59], which increases only in males. Thus, in the junco, the sexes differ in their phenotypic response to T for some, but not all traits. We hypothesize that this difference may arise partly because of differences in transcriptional response to T-treatment.

Direct measurement of survival and reproductive success in free-living juncos also suggests that the sexes may differ in the fitness consequences of T [39], [47], [60]. In males, experimental elevation of T reduces survival, but the reduction is more than offset by an increase in extra-pair mating success [39], [61] with the result that fitness in T-treated males is greater than that of controls [39]. These results suggest that selection would favor males with higher T if such males were to occur naturally. However, females treated with T have been shown to have lower fitness than controls [47], [60], suggesting that an elevation in T in females resulting from an evolutionary response to selection on males could be detrimental to females [17]. This dynamic is consistent with sexual conflict in which the negative fitness consequences of higher T in females might constrain the response of males to selection favoring higher T [17], [39].

To address the role of hormone-mediated and sexually dimorphic transcriptional response in accounting for the behavioral effects of T, we analyzed gene expression in two brain regions related to these effects: the medial amygdala and the hypothalamus. The medial amygdala is associated with many social behaviors, including sexually dimorphic aggressive and reproductive behaviors in birds and rodents [62]–[65]. The hypothalamus regulates several aspects of homeostasis, hormone balance, and seasonal behavior [66]–[69]. Both the medial amygdala and the hypothalamus express high levels of androgen receptors [62], [70], and are thus likely to respond to experimental manipulation of T. Importantly, these brain areas are also major sites of estrogenic action, and many of the effects of sex steroids in these regions may occur after local conversion of T to estradiol [71], [72]. Furthermore, the medial amygdala plays a key role in relaying and mediating social signals between brain areas [73], and the hypothalamus is a major control center of hormones and behavior with projections extending throughout the brain [74]. Thus, transcriptional changes in these brain areas are likely to reflect not just the direct effect of sex steroids, but are also likely to be indirectly affected by T-induced behavioral changes and T-induced changes in other areas of the brain and body as well.

We first asked how baseline gene expression differed between the sexes. We predicted that control males and control females would differ in the expression of key genes related to known sex differences, such as sexual and social behavior. We then assessed the impact of experimental elevation of T within each sex. Here we predicted that T-treatment would influence genes related to traits known to respond to T-treatment in both sexes, such as immune function, metabolism, and several behaviors. Because some traits are insensitive to T in one sex, we also predicted that T would affect the sexes in contrasting ways, such that at least some of the genes that were differentially expressed between T-treated individuals and controls would differ by sex.

## Materials and Methods

### Ethics Statement

This study was carried out in strict accordance with all regulations and guidance of the Guide for the Care and Use of Laboratory Animals of the National Institutes of Health. All animal methods were reviewed and approved by the Institutional Animal Care and Use Committee at Indiana University–Bloomington (Protocol #09-037). All implants were performed with local anesthetic and euthanasia was conducted in accordance with the AVMA Guidelines on Euthanasia. Animals were captured near Mountain Lake Biological Station on publicly accessible roadways in and around Jefferson National Forest. Animal collection permits were obtained from the: U.S. Department of the Interior (Permit Number: 20261), U.S. Fish and Wildlife Service (Permit Number: MB093279-0), and Virginia Department of Game and Inland Fisheries (Permit Number: 041506).

### Animal collection and treatment

We collected 26 adult dark-eyed juncos (14 male, 12 female) from breeding grounds near Mountain Lake Biological Station (Pembroke, VA; 37° 22′ 31″N, 80° 31′ 24″W). Individuals were captured in mist-nets during the early breeding season (7 to 14 May 7 2010) and held individually in a semi-naturalistic outdoor aviary. Each individual had its own compartment (0.60×1.12×2.38 m). Animals were not acoustically or visually isolated from each other.

Following capture, individuals were treated with implants of silastic tubing that were either empty (control) or packed with crystalline T (Sigma-Aldrich, St. Louis, Missouri, USA). Males treated with T received two 10 mm implants, while females received a single 5 mm implant. These implants have been used historically in the study of the junco and have been repeatedly shown to yield physiological maximum levels of T in each sex [17]. We placed all implants subcutaneously along the right flank of the bird with a trochar needle under local anesthetic on May 14 and 15. Implants were checked the following day and again at the time of euthanasia to ensure proper placement. In all, four treatment groups were created: control males (n = 7), testosterone-treated males (n = 7), control females (n = 6), and testosterone-treated females (n = 6). Six individuals (males were randomly selected for inclusion) from each treatment group were used to analyze each tissue.

The specifics of our implant regimen were chosen to mimic previous studies and to capture the largest possible range of effects of T-treatment, including both direct and indirect effects. Testosterone is aromatizable, and many of the effects of T are known to be mediated by local conversion of T to estradiol [75]. Similarly, the duration of the implant exposure (26 days) is sufficient to establish stable phenotypic effects [37], [53], and to allow for both direct and indirect phenotypic effects of T-treatment on tissues and gene expression. Thus, we note that many of the effects of T-treatment, both phenotypic in previous studies and transcriptional in this study, are likely to be indirect stemming from conversion of T to other hormones, the interaction of T with other signaling systems, and feedback from behavioral and physiological changes directly induced by T-treatment. These direct and indirect effects reflect the natural response of the organism to elevated T levels and to the T-implants utilized in previous studies.

### Tissue collection and RNA extraction

On June 9 and 10, 26 days after treatment, individuals were euthanized by overdose of isoflurane. Sacrifices occurred between the hours of 0700 and 1230. Sexes and treatments were balanced across days and time of day due to the potential for circadian changes in expression of some genes. Tissues, including whole brains, were collected rapidly (within 20 minutes post-mortem) and stored on powdered dry ice to ensure negligible RNA degradation [76]. Brains were later dissected into 14 distinct regions using anatomical landmarks, following previously established methods [77], [78]. Briefly, each brain was placed onto a sterile, chilled glass petri dish over ice and allowed to thaw only enough to permit microdissection. After removing optic chiasm, optic tecta, and the hindbrain, we collected the diencephalon to the depth of the anterior commissure. This dissection includes the most rostral portion of the thalamus, but is largely limited to the hypothalamus [77], and so it will be referred to as hypothalamus throughout the manuscript. We then removed approximately 1 mm of the ventromedial portion of the caudal telencephalon, which is largely limited to the medial amygdala [72]. The newly separated regions were rapidly returned to −80°C. RNA was later extracted in TRIzol, following manufacturer directions (Invitrogen, Carlsbad, CA, USA). All RNA was high quality as measured by Agilent Bioanalyzer (Santa Clara, CA, USA) with RNA integrity number [79] scores ranging from 7.6–9.4.

### Microarray platform

Gene expression was analyzed using a custom microarray for the dark-eyed junco based on transcriptome sequencing [80]. Briefly, this Nimblegen 12-plex microarray (Roche Nimblegen, Inc., Madison, WI) contained 100,635 features representing 33,545 contigs (assembled sequencing reads) in triplicate covering 22,765 isogroups (putative genes). An additional 34,365 probes singly representing unassembled singletons were omitted from this analysis. Annotation was accomplished by sequence similarity against the NCBI non-redundant protein database [81] using blastx for gene identities and Blast2GO [82] for functional annotation with gene ontology (GO) terms [83].

### cDNA preparation and hybridization

Microarray experiments were conducted as described in [80] following [84]. Briefly, we performed double strand cDNA synthesis using the Invitrogen SuperScript Double-Stranded cDNA Synthesis kit with random hexamer primers and labeled cDNA using 1 O.D. CY-labeled random nonamer primer (either Cy3 or Cy5) and 100 U Klenow fragment per 1 µg ds-cDNA (following NimbleGen labeling protocols). We then hybridized 15 µg of each of two labeled samples (one Cy3, one Cy5) to each sub-array, following a full round robin design within each tissue (n = 6 per treatment group for each tissue), and followed manufacturer's directions for post-hybridization washing and scanning (Roche NimbleGen, Inc., Madison, WI). Imaging was accomplished by Axon GenePix 4200A scanner (Molecular Devices, Sunnyvale CA) with GenePix 6.0 software, and data were extracted with NimbleScan 2.4 (Roche NimbleGen, Inc., Madison WI). Raw microarray data were processed and normalized with the limma package [85] in R [86]. The microarray data are available in the NCBI Gene Expression Ominubus repository (accession number: GSE41076).

### Microarray analysis

Following normalization, we identified contigs that were expressed in each condition by comparing contig expression scores to randomized probes, as described in [80]. Using only contigs expressed in at least one of the compared treatment groups, we made three comparisons for each tissue using the limma package [85] of bioconductor in the R statistical package [86]: control males vs. control females; control males vs. testosterone-treated males; and control females vs. testosterone-treated females (n = 6 per treatment group for each tissue).

For calculations, statistics, and visualization, we used the log_2_ fold change between sexes or between treatment groups, along with the modified t-statistic and p-value, calculated in the limma package for most isogroups. However, for isogroups represented by more than one contig (4,288 of 22,765 isogroups), we calculated the mean t-value of all contigs and calculated significance on degrees of freedom equal to the total number of probes scored for the isogroup minus two. The median fold change from its representative contigs was assigned to each isogroup. To reduce false-positives, we set a false discovery threshold of 0.05 [87] and calculated global q-values (FDR control for all tissues and comparisons) using the R package qvalue [88].

We then used topGO [89] to identify the GO terms that were significantly over-represented among the significantly differentially expressed genes in each comparison using the weight algorithm [90], Fisher's exact test, and a p-value cut-off of 0.05. GO terms with fewer than five annotations were not analyzed, and terms with over-representation driven by fewer than 3 genes were not reported.

## Results

### Sex differences

**Figure 1:**
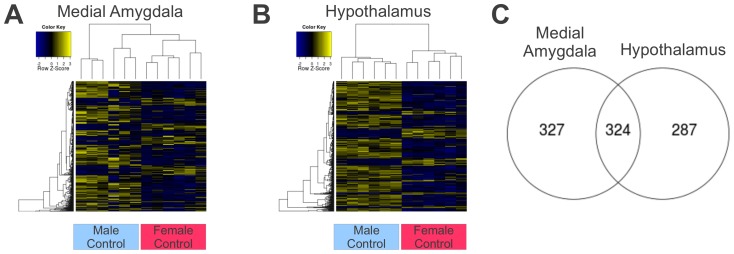
Sex differences in gene expression. Differences in gene expression between the sexes are represented by heat maps that show scaled individual expression scores for just the significantly differentially expressed genes in the medial amygdala (a) and hypothalamus (b). Venn diagram shows the overlap in significant genes between the two tissues (c).

We identified many genes as significantly differentially expressed between control males and control females in each brain region. In the medial amygdala 651 isogroups (of 15,254 expressed) were differentially expressed between the two sexes (Figure 1a) with 476 higher in control males than females (Table S1 in file S1) and 175 higher in control females than males (Table S2 in file S1). Here, GO analysis identified 46 terms that were over-represented among the significantly differentially expressed genes (Table S3 in file S1), including the terms: *microtubule cytoskeleton*, *positive regulation of synaptic plasticity by chemical substance*, *positive regulation of cell growth*, *neuron projection*, *RAS protein signal transduction*, *and cytokinesis*.

In the hypothalamus, 611 isogroups (of 15,624 expressed) were differentially expressed by sex (Figure 1b) with 483 higher in control males than females (Table S4 in file S1) and 128 higher in control females than males (Table S5 in file S1). Among these 611 genes, GO analysis identified 60 terms that were significantly over-represented (Table S6 in file S1). These GO terms included: regulation of *microtubule cytoskeleton*, *positive regulation of synaptic plasticity by chemical substance*, *positive regulation of cell growth*, *axon regeneration*, *and sterol metabolic process*.

Among theses genes, 324 genes were significantly differentially expressed between the sexes in *both* medial amygdala and hypothalamus (Figure 1c). Of these genes, 253 were higher in control males than control females in both tissues (Table S7 in file S1) and 71 were higher in control females than control males in both tissues (Table S8 in file S1). None was differentially expressed in opposite directions in the tissues.

### Effect of T-treatment in females

**Figure 2:**
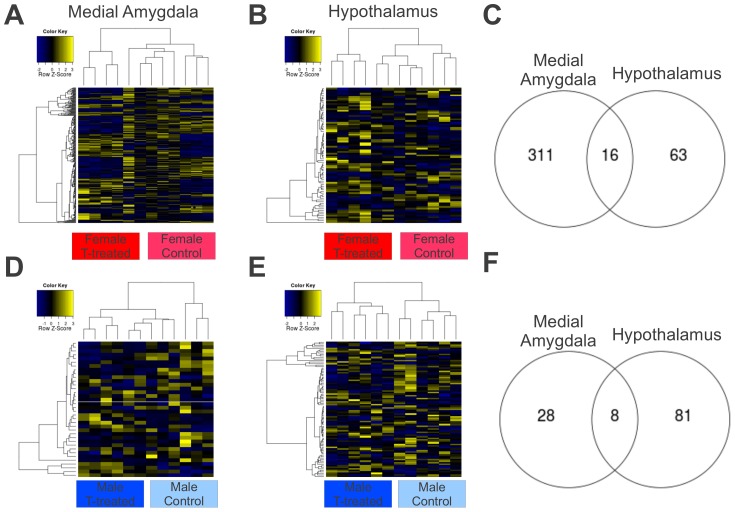
Gene expression in response to T-treatment in each sex. Differences in gene expression between T-treated and control individuals in both the medial amygdala (left column) and the hypothalamus (middle column) for response to females (a–c) and in males (d–fh). Heat maps show scaled individual expression scores for just the genes that were significantly differentially expressed between T-treated and control individuals in each sex (a,b,d,e). Venn diagram shows the overlap of significant within each contrast between the tissues. See text and supplementary tables for more information.

In both brain regions, there were significant differences in expression between T-treated and control females. In the medial amygdala, 327 isogroups (of 15,110 expressed) were significantly differentially expressed between T-treated and control females (Figure 2a) with 167 higher in T-treated than control females (Table S9 in file S1) and 160 lower in T-treated than control females (Table S10 in file S1). GO analysis identified 18 over-represented terms (Table S11 in file S1), including: *microtubule polymerization or depolymerization*, *structural molecule activity*, and *ribosome*.

In the female hypothalamus, 79 isogroups (of 15,546 expressed) were differentially expressed (Figure 2b) with 49 higher in T-treated than control females (Table S12 in file S1) and 30 lower in T-treated than control females (Table S13 in file S1). GO analysis identified 15 over-represented terms (Table S14 in file S1), including: *metal ion binding*, *detection of chemical stimulus*, *and response to nutrient levels*.

In females, 16 genes were significantly differentially expressed between T-treated and control individuals in *both* medial amygdala and hypothalamus (Figure 2c). Among these genes, 9 were higher (Table S15 in file S1) and 7 were lower (Table S16 in file S1) in T-treated than control females in both tissues. No genes were significantly affected in opposite directions in the two tissues.

### Effect of T-treatment in males

In the medial amygdala, 36 isogroups (of 15,279 expressed) were significantly differentially expressed between T-treated and control males (Figure 2d) with 15 higher in T-treated than control males (Table S17 in file S1) and 21 lower in T-treated than control males (Table S18 in file S1). GO analysis identified four over-represented terms: *metal ion transport, ion channel activity, cation transmembrane transporter activity*, and *integral to membrane*.

In the male hypothalamus, 89 isogroups (of 15,540 expressed) were significantly differentially expressed between treatment groups (Figure 2d) with 35 higher in T-treated than control males (Table S19 in file S1) and 54 lower in T-treated than control males (Table S20 in file S1). GO analysis identified one over-represented term: *phosphatase activity*.

In males, 8 genes were differentially expressed between T-treated and control individuals in both medial amygdala and hypothalamus (Figure 2c). Among these genes, 4 were higher (Table S21 in file S1) and 4 were lower (Table S22 in file S1) in T-treated than control males in both tissues. No genes were significantly affected in opposite directions in the two tissues.

### Comparing the effect of T-treatment in the sexes

In both the medial amygdala and hypothalamus, a small number of genes were differentially expressed between T-treated and control individuals of both sexes. Three genes were significantly differentially expressed between T-treated and control individuals of both sexes in the medial amygdala (t*ransient receptor potential cation channel*, *subfamily M*, *member 8*, and two unannotated genes), and they were each lower in T-treated than control males, but higher in T-treated than control females. One gene (*Cytochrome P450 19A1*) was significantly differentially expressed between T-treated and controls of both sexes in the hypothalamus, and that gene was more highly expressed in T-treated than control individuals in both sexes.

## Discussion

We identified many genes that were differentially expressed between control males and females and between T-treated and control individuals of each sex. As predicted, the affected genes were often related to known sexual dimorphisms and previously described effects of T-treatment on phenotype (elaborated below). However, there was a substantial difference in response to T-treatment in males and females: T-treatment influenced the expression of different sets of genes in each sex in both tissues. The difference between the sexes in hormonally regulated gene expression is a key to understanding sexual dimorphism and sexual conflict, and the specific genes identified here may mediate some of these processes.

### Sexually dimorphic gene expression

Similarly to other studies (reviewed in [18]), we identified a substantial number of genes that were expressed differentially in males and females. In general, our results resembled patterns found in previous comparisons of sex-biased gene expression in whole brains of other song birds [19], although we identified a greater number of sexually dimorphic genes (611 in the hypothalamus and 651 in the medial amygdala) than were reported in the whole brain of zebra finch and common whitethroat (*Sylvia communis*; 509 and 345, respectively; [19]). This difference may relate to fact that we analyzed two specific brain regions as opposed to whole brain. Others have noted that pooling tissues comprised of discrete regions can reduce ability to detect differences [65], likely because changes in gene expression in particular regions may be masked if the expression level was modified in only a subset of tissues that were analyzed collectively. Significant gene expression differences between fine scale regions of the human [91] and bird [92] brain suggest that sex differences in gene expression may vary significantly between regions as well.

The consensus from comparative neurobiology suggests that social stimuli are relayed through the medial amygdala to modify how animals respond to social stimuli, and many of the behaviors influenced by the medial amygdala are sexually dimorphic, including social [93] and aggressive [94] behaviors. Thus, it is not surprising that among the differentially affected genes, there were several receptors that have been directly related to behavior in model systems. For example, *galanin receptor 3* (*GALR3*) was expressed more in males than females, and galanin, its ligand, has been implicated in several clinical conditions that are known to affect one sex more than another, including depression and anxiety [95], [96] and human alcoholism [97].

In the hypothalamus, we found differential expression of a number of cholesterol- and steroid-related genes, consistent with the role of the hypothalamus in regulating steroid levels. Specifically, *HMG-CoA reductase*, *HMG-CoA synthase*, and *hydroxysteroid (17-beta) dehydrogenase 4* (*HSD17B4*), were all more highly expressed in control males than control females. *HMG-CoA reductase*, and *HMG-CoA synthase*, are key enzymes in the production of cholesterol via the mevalonate pathway [98], a necessary step for *de novo* production of steroid hormones. *HSD17B4*, is involved, primarily, in the oxidative breakdown of estradiol [99]. Combined, these findings suggest that males may be producing more steroids (or their precursors), but also more rapidly turning them over, compared to females. In addition, *follistatin* was more highly expressed in control males than control females. *Follistatin* plays a wide-range of roles throughout the body [100]; however, in the brain it suppresses the release of follicle stimulating hormone, a key regulator of gametogenesis [101], from the pituitary [102]. Together, the differential expression of these genes is consistent with the known role of the hypothalamus in regulating hormonal and reproductive physiology differently in males and females.

Results in mammals find a limited number of genes with sexually dimorphic expression in the brain as compared to other tissues, and these genes often appear to have potentially large downstream effects [103], as do the genes identified in this study. For example, in mice, *RNA helicase activity* is the only function over-represented among genes differentially expressed in brain between males and females [65]. In other species, many of the genes and functions identified as differentially expressed between males and females relate to translation and suggest large downstream effects that cannot be identified by gene expression analysis [103]. In our study, several transcription factors (nine in the medial amygdala and six in the hypothalamus) were differentially expressed between control males and females. If these transcription factors have a subtle effect on expression of other genes, then perhaps they affected the expression of many other genes, but below the level of detection of this experiment. Many of the genes we identified as differentially expressed by sex were also related to cell growth and cytoskeleton structure. For instance, genes related to microtubule formation were expressed at higher levels in males than in females in both the medial amygdala and hypothalamus, perhaps suggesting that males were more actively maintaining and remodeling the cellular structure of these brain regions.

### Effect of T-treatment in females

Several of the genes that were differentially expressed between T-treated and control females have been linked to aggressive behavior in the past, suggesting a connection with the known effect of T-treatment on aggression in juncos [43] and other species [28], [52]. *Cytochrome P450 19A1*, the aromatase responsible for the enzymatic conversion of T to estradiol [71], was more highly expressed in the hypothalamus of T-treated than control females. Local metabolism of T into estradiol is known to meditate many of the well known effects of T [75], suggesting that the higher hypothalamic expression of aromatase may mediate some of the behavioral effects of T-treatment. Neural aromatase expression and activity is associated with sexual [104] and aggressive behavior [105] and correlates with aggression in juncos [78]. Similarly, *monoamine oxidase A* (*MAO-A*) is less expressed in the medial amygdala of T-treated than control females. *MAO-A* degrades both dopamine and serotonin, and decreased or absent functioning of *MAO-A* increases aggression in mice [106] and humans [107]. Thus, expression changes in *Cytochrome P450 19A1* and *MAO-A* may partially mediate the effects of T-treatment on aggression.

Another set of differentially expressed genes appears to be related to the metabolic and activity effects of T-treatment [59], [108], [109]. *Cannabinoid receptor 1* (*CB_1_*) was more highly expressed in the hypothalamus of T-treated than control females. This is one of the genes annotated with the GO term *response to nutrient levels*, and the role of *CB_1_* in signaling hunger [110] may be involved in the reduced body mass and fattening induced by T-treatment [45], [56]. Further, *GALR3*, which can affect activity [95], [111], is less expressed in medial amygdala of T-treated than control females.

Although we did not directly measure phenotypes in this study, more than a dozen previous studies in juncos have demonstrated that this same T-treatment masculinizes several female behaviors [17], including reduced nest defense [47], increased aggression [43], and reduced mate choosiness [112], to levels more similar to males [33], [113], [114]. Findings in other species also support a role for T in masculinizing female behavior and brain morphology [16], [17], [25], along with gene expression [22], [23], [115]. In both sexes, many of the neural effects of testosterone are actually mediated by estradiol, after testosterone is locally converted via aromatase [75]. Further, if T-treatment directly caused a behavioral change, then gene expression in the brain may be a response to that modified behavior, rather than a direct response to T. Thus, we cannot distinguish whether the gene expression effects we quantified were the direct effect of T or caused by these indirect routes; however, these mechanisms likely operated in previous studies of T-treatment and therefore reflect the transcriptional changes related to known phenotypic effects of T-treatment.

### Effect of T-treatment in males

Many of the genes identified as differentially expressed between T-treated and control males are related to previously identified phenotypic effects of T-treatment. Further, it appears that several of the differentially expressed genes impact signaling systems that are likely to have large influence on a number of phenotypes. Testosterone is a hormone that has pleiotropic effects on organismal phenotype [36]; however it is possible that these sweeping effects are *not* the result of T-treatment affecting the expression of many different genes (either directly or indirectly), but rather the result of T-treatment altering the expression of only a few genes with major pleiotropic effects on a broad array of phenotypes.

Several genes appear to be related to both the aggressive- [55] and activity- [59] related effects of T-treatment. For example, in hypothalamus, *cytochrome P450 19A1*, the aromatase responsible for the enzymatic conversion of T to estradiol [71], was more highly expressed in T-treated than control males. As in females (see above), change in expression of aromatase may explain several aspects of the aggressive and sexual response to T-treatment in juncos [75], [78], [116]. In mice and humans, decreased *melanocortin 4 receptor* (*MC4R*) activity increases feeding and obesity [117]–[119]. Thus, higher expression of *MC4R* in hypothalamus of T-treated than control males is consistent with previous findings of reduced body mass following T-treatment in songbirds [45], [56], though it is important to note that such an effect could come about because MC4R was directly affected by T-treatment or because T-treatment induced changes in feeding and metabolism that altered expression of MC4R.

Thus, it is possible that the expression change in these few genes could account for a large proportion of the previously described phenotypic effects of T-treatment. As another example, *MGC89063* protein, a major transcriptional cofactor [83], [120], was expressed more highly in T-treated than control males in the hypothalamus and medial amygdala. *MGC89063* may play a wide role in modifying gene expression, and if its downstream effects on gene-expression were numerous but small or in different brain regions, they may be below the level of detection for microarray experiments yet still be biologically meaningful [121].

### Comparing the effect of T-treatment in the sexes

Most of the genes identified as significantly differentially expressed between T-treated individuals and controls in one sex were not also significant in the other sex, suggesting that different genes were being affected by T-treatment in each sex. The small overlap between genes that were affected by T-treatment in males and females is particularly puzzling, because T-treatment elicits many of the same behavioral and physiological outcomes in both sexes (reviewed in [17]). Our data suggest that the sexes may arrive at particular phenotypic outcomes via transcriptional changes in different genes, though other systems give reason to doubt the generality of this finding. For example, dominant social behavior in cichlid fish appears to be due to the same gene expression mechanisms in both males and females [122], demonstrating that similar phenotypes in males and females may be arrived at through the same transcriptional mechanisms as well.

Among the genes that were significantly differentially expressed between T-treated and control individuals in *both* sexes, there was no clear pattern relating the sexes. In the hypothalamus, *cytochrome P450 19A1* (aromatase) was higher in T-treated than control individuals of both sexes, possibly due to its role in local conversion of T to estradiol, which may be the mediator of some of the known effects of T-treatment (see above for descriptions in males and females). In the medial amygdala, the three genes that were differentially expressed between T-treated and control individuals were all higher in T-treated than control in females and lower in T-treated than control in males. Only one of these, (*transient receptor potential cation channel*, *subfamily M*, *member 8*; *TRPM8*) was annotated. *TRPM8* responds to cold stimuli in sensory cells [123], but whether it mediates known phenotypic effects of T-treatment via the medial amygdala is unclear.

It appears that the similar phenotypic outcomes of T-treatment described in previous studies may be caused by expression changes in different genes in each sex. For example, activity and metabolism appear to be affected by T-treatment in multiple species (e.g., [109], [124]), but in juncos, only males, not females, increase their home-range size in response to T-treatment [58], [59]. As described above, it appears that some of these metabolic and activity effects of T-treatment [109], [124] could be mediated by changes in expression of *CB_1_* and *GALR3* in females [125], [126], while *MC4R* expression may mediate this effect in males [118]. Perhaps these different transcriptional routes are a mechanism that has allowed for the divergence of male and female response to T-treatment.

The difference in T-dosage given to each sex may also relate to the differential response to T-treatment in males and females. We administered one 5 mm implant to females to induce T-levels at the high end of the natural distribution of female T, but because male T is naturally much higher, we used two 10 mm implants to induce T-levels at the high end of the natural distribution of male T. Thus, while these doses were sex-appropriate [17] and match the doses used when measuring phenotypes in previous studies (e.g. [46], [112]), it remains possible that there is a bell-shaped dose-response curve to T such that T-treatment in males suppressed the expression of genes that were enhanced by T-treatment in females. Thus, it is possible that had we treated females with the same dose as males, the responses might have been similar. However, we do not think this explanation is the most parsimonious because these same dosages of T implant induce similar behavior and physiology in male and female juncos (summarized above). Further, identical doses of T would not ensure that the sexes experience the implants in the same way. For example, in wintering juncos given identical 5-mm T-implants, females had circulating levels of T that were significantly higher than males [127], suggesting that male and female processing of exogenous T may differ.

The fact that both males and females have been shown to respond phenotypically to T-treatment indicates the potential for sexual antagonism over the optimal circulating level of hormones [24]–[26]. However, it is also known that females are not sensitive to all of the same behavioral and physiological effects of hormonal treatment as males (reviewed in [17]). Female insensitivity to T-treatment with respect to some phenotypes suggests that, in some species, the sexes may process or interpret a hormonal signal differently, which is consistent with our finding that T-treatment affects different genes in each sex. Thus, not only do the sexes differ in naturally circulating levels of T and phenotypic response to T-treatment [17], but, as we have shown here, they also differ in the downstream genomic effects seen in response to experimental treatment with T. It is possible that the sex difference in transcriptional response to T-treatment could be a consequence of additional modulators of gene expression that control the way in which T interacts with the genome (e.g. androgen receptor co-activators or DNA methylation patterns). Sexual dimorphism in the genes that are affected by T-treatment may be a key step in resolving sexual conflict over optimal circulating T levels [24]–[26] by modifying the phenotypic effect of T separately in each sex.

## Conclusions

In this study, we applied genomic tools and functional knowledge from model systems to a species with well-studied ecology to gain novel insights into sexual dimorphism and hormones in a natural system. We identified a large number of genes that are likely to play specific roles in both sexual dimorphism and the behavioral effects of testosterone. Further investigation is warranted to determine whether these differences in gene expression contributed to the previously identified phenotypic effects of T-treatment and to assess the mechanisms relating T-treatment, gene expression, and phenotypic effects. Specifically, RNAi knockdown of these genes would allow for analysis of the immediate impact of targeted changes in gene expression on behavior. T-treatment affected largely different genes in males and female, suggesting that T-treatment may bring about similar behavioral and physiological effects on the sexes by different transcriptional mechanisms, potentially opening a route to the reduction of sexual conflict over optimal levels of T. We were especially intrigued by the possibility that only a few genes in a few tissues may mediate the pleiotropic phenotypic effects previously observed in response to T-treatment.

## Supporting Information

File S1 **Tables S1–S22 list the genes that were significantly differentially regulated in each of the tested comparisons and the GO terms that were over-represented.**
(PDF)
