# Supplementary material for: Testosterone Affects Neural Gene Expression Differently in Male and Female Juncos: A Role for Hormones in Mediating Sexual Dimorphism and Conflict
Source: PLoS One. 2013 Apr 16;8(4):e61784. doi: 10.1371/journal.pone.0061784 (PMC3627916; doi:10.1371/journal.pone.0061784)
Supplement: File S1 — Tables S1–S22 list the genes that were significantly differentially regulated in each of the tested comparisons and the GO terms that were over-represented. (PDF) [file pone.0061784.s001.pdf]

Supplementary Table 1. Genes expressed higher in males than females in the medial amygdala.

Isogroup	Annotation from nr Database
isogroup00092	PREDICTED: spectrin, beta, non-erythrocytic 1 [Taeniopygia guttata]
isogroup00224	PREDICTED: chromosome 9 open reading frame 3 [Taeniopygia guttata]
isogroup00277	chromo-helicase DNA-binding protein [Zonotrichia albicollis]
isogroup00337	PREDICTED: hypothetical protein [Gallus gallus]
isogroup00365	PREDICTED: similar to DKFZP459P083 protein isoform 1 [Taeniopygia guttata]
isogroup00451	PREDICTED: similar to GRAM domain containing 3 [Taeniopygia guttata]
isogroup00458	SWI/SNF related, matrix associated, actin dependent regulator of chromatin, subfamily a, member 2 [Gallus gallus]
isogroup00524	PREDICTED: hypothetical protein [Taeniopygia guttata]
isogroup00530	PREDICTED: similar to Uncharacterized protein C9orf85 [Taeniopygia guttata]
isogroup00533	DNA-directed DNA polymerase kappa [Gallus gallus]
isogroup00551	RPTOR independent companion of MTOR, complex 2 [Bos taurus]
isogroup00570	single-stranded DNA binding protein 2 [Homo sapiens]
isogroup00574	PREDICTED: similar to semaphorin 5A [Taeniopygia guttata]
isogroup00606	NA
isogroup00694	PREDICTED: prolactin receptor [Taeniopygia guttata]
isogroup00744	PREDICTED: xeroderma pigmentosum, complementation group A [Taeniopygia guttata]
isogroup00779	PREDICTED: SMC5 protein [Taeniopygia guttata]
isogroup00783	PREDICTED: death-associated protein kinase 1 [Taeniopygia guttata]
isogroup00794	PREDICTED: similar to Lipid phosphate phosphohydrolase 1 [Taeniopygia guttata]
isogroup00897	COP9 constitutive photomorphogenic homolog subunit 6 [Xenopus laevis]
isogroup00941	PREDICTED: similar to plasma membrane calcium ATPase 2 isoform 2 [Gallus gallus]
isogroup00984	PREDICTED: similar to KIAA0372 [Gallus gallus]
isogroup00991	PREDICTED: jumonji domain containing 2C [Taeniopygia guttata]
isogroup00999	PREDICTED: COBW domain containing protein1 [Taeniopygia guttata]
isogroup01029	PREDICTED: alpha2,3-sialyltransferase VI isoform 1 [Taeniopygia guttata]
isogroup01050	PREDICTED: zinc finger, CCHC domain containing 6 [Taeniopygia guttata]
isogroup01057	PREDICTED: similar to 1-phosphatidylinositol-4,5-bisphosphate phosphodiesterase beta-4 isoform 1 [Taeniopygia guttata]
isogroup01062	syntaxin 6 [Gallus gallus]
isogroup01070	NA
isogroup01106	PREDICTED: similar to SHC (Src homology 2 domain containing) transforming protein 3 [Taeniopygia guttata]
isogroup01149	macrophage receptor with collagenous structure [Gallus gallus]
isogroup01181	PREDICTED: similar to phosphoinositide-3-kinase, class 3 [Taeniopygia guttata]

Isogroup	Annotation from nr Database
isogroup01284	PREDICTED: RAS p21 protein activator (GTPase activating protein) 1 [Taeniopygia guttata]
isogroup01320	PREDICTED: similar to SMC1 structural maintenance of chromosomes 1-like 1 isoform 2 [Macaca mulatta]
isogroup01374	NA
isogroup01390	NA
isogroup01394	PREDICTED: similar to KIAA1066 protein [Gallus gallus]
isogroup01405	PREDICTED: G patch domain containing 8 [Taeniopygia guttata]
isogroup01427	PREDICTED: microtubule-associated protein 1B isoform 1 [Taeniopygia guttata]
isogroup01461	PREDICTED: lysosomal trafficking regulator [Taeniopygia guttata]
isogroup01466	PREDICTED: similar to protein geranylgeranyltransferase type I, beta subunit isoform 1 [Equus caballus]
isogroup01658	Rpl17 protein [Mus musculus]
isogroup01772	perilipin 1 [Gallus gallus]
isogroup01805	SOSS complex subunit C [Gallus gallus]
isogroup01811	PREDICTED: survival of motor neuron [Taeniopygia guttata]
isogroup01868	PREDICTED: ankyrin repeat domain protein 15 [Gallus gallus]
isogroup01902	PREDICTED: NADH dehydrogenase (ubiquinone) Fe-S protein 4 [Taeniopygia guttata]
isogroup01948	PREDICTED: ST8 alpha-N-acetyl-neuraminide alpha-2,8-sialyltransferase 5 [Taeniopygia guttata]
isogroup02203	PREDICTED: transmembrane protein 161B [Taeniopygia guttata]
isogroup02212	PREDICTED: ubiquitin specific protease 4 [Taeniopygia guttata]
isogroup02238	PREDICTED: similar to putative peptidylprolyl isomerase C [Taeniopygia guttata]
isogroup02286	NA
isogroup02410	Coiled-coil domain containing 101 [Mus musculus]
isogroup02530	PREDICTED: ankyrin repeat domain 32 [Taeniopygia guttata]
isogroup02549	NA
isogroup02560	unnamed protein product [Homo sapiens]
isogroup02714	PREDICTED: similar to LOC100170471 protein [Taeniopygia guttata]
isogroup02725	neurexin 3 beta precursor [Gallus gallus]
isogroup02754	PREDICTED: v-abl Abelson murine leukemia viral oncogene homolog 2 (arg, Abelson-related gene) [Taeniopygia guttata]
isogroup02775	PREDICTED: similar to synaptic vesicle glycoprotein 2b [Taeniopygia guttata]
isogroup02806	NA
isogroup02941	PREDICTED: similar to mesoderm induction early response 1, family member 2 [Taeniopygia guttata]
isogroup02948	PREDICTED: hypothetical protein [Taeniopygia guttata]

Isogroup	Annotation from nr Database
isogroup02951	PREDICTED: similar to RAD26L hypothetical protein [Equus caballus]
isogroup02999	PREDICTED: solute carrier family 44, member 2, partial [Taeniopygia guttata]
isogroup03045	PREDICTED: similar to zinc finger protein 423 [Gallus gallus]
isogroup03074	PREDICTED: multiple PDZ domain protein [Taeniopygia guttata]
isogroup03097	PREDICTED: phosphatidylinositol-4-phosphate 5-kinase, type I, beta [Taeniopygia guttata]
isogroup03129	PREDICTED: ring finger protein 20 [Taeniopygia guttata]
isogroup03176	PREDICTED: similar to polyadenylate binding protein-interacting protein 1 [Taeniopygia guttata]
isogroup03256	PREDICTED: similar to KIF27A [Monodelphis domestica]
isogroup03336	PREDICTED: PC4 and SFRS1 interacting protein 1 [Taeniopygia guttata]
isogroup03347	AF211859_1 cyclin ania-6b [Mus musculus]
isogroup03383	PREDICTED: similar to UBQLN1 protein [Gallus gallus]
isogroup03391	NA
isogroup03406	PREDICTED: similar to ELAV (embryonic lethal, abnormal vision, Drosophila)-like 2 (Hu antigen B) (AKA Mel-N1), partial [Gallus gallus]
isogroup03418	NA
isogroup03443	PREDICTED: similar to protein-L-isoaspartate (D-aspartate) O-methyltransferase domain containing 2 [Ornithorhynchus anatinus]
isogroup03489	PREDICTED: similar to myosin IXA [Taeniopygia guttata]
isogroup03572	PREDICTED: arrestin domain containing 3 [Taeniopygia guttata]
isogroup03582	PREDICTED: etoposide induced 2.4 mRNA [Taeniopygia guttata]
isogroup03596	PREDICTED: adenomatous polyposis coli [Taeniopygia guttata]
isogroup03607	PREDICTED: zinc finger protein 533 [Taeniopygia guttata]
isogroup03644	PREDICTED: similar to KIAA0833 protein [Rattus norvegicus]
isogroup03651	PREDICTED: hypothetical protein [Gallus gallus]
isogroup03694	PREDICTED: similar to MEK kinase 1 [Gallus gallus]
isogroup03703	NA
isogroup03724	PREDICTED: family with sequence similarity 102, member A [Taeniopygia guttata]
isogroup03789	PREDICTED: hypothetical protein [Taeniopygia guttata]
isogroup03812	NA
isogroup03930	PREDICTED: prion protein interacting protein [Taeniopygia guttata]
isogroup03949	PREDICTED: similar to Mahogunin, ring finger 1 [Gallus gallus]
isogroup03958	SWI/SNF related, matrix associated, actin dependent regulator of chromatin, subfamily a, member 2 [Gallus gallus]
isogroup03976	PREDICTED: similar to inositol 1,4,5-trisphosphate 3-kinase B [Taeniopygia guttata]
isogroup04049	PREDICTED: similar to semaphorin SEMA6A1 [Gallus gallus]

Isogroup	Annotation from nr Database
isogroup04163	PREDICTED: acetyl-coenzyme A acyltransferase 2 [Taeniopygia guttata]
isogroup04199	PREDICTED: putative clathrin light polypeptide variant 1a [Taeniopygia guttata]
isogroup04207	NA
isogroup04217	PREDICTED: similar to P450 (cytochrome) oxidoreductase [Taeniopygia guttata]
isogroup04227	PREDICTED: similar to DnaJ (Hsp40) homolog, subfamily B, member 5 [Taeniopygia guttata]
isogroup04250	PREDICTED: putative sorting nexin 24 [Taeniopygia guttata]
isogroup04483	PREDICTED: hypothetical protein [Taeniopygia guttata]
isogroup04504	NA
isogroup04531	NA
isogroup04581	NA
isogroup04720	PREDICTED: non-imprinted in Prader-Willi/Angelman syndrome 1 [Taeniopygia guttata]
isogroup04723	PREDICTED: WD repeat domain 70 [Taeniopygia guttata]
isogroup04781	NA
isogroup04828	PREDICTED: cholinergic receptor, nicotinic, beta polypeptide 3 [Taeniopygia guttata]
isogroup04836	PREDICTED: similar to transcription factor 20 [Gallus gallus]
isogroup04877	hypothetical protein PANDA_011833 [Ailuropoda melanoleuca]
isogroup04886	NA
isogroup04905	PREDICTED: similar to Chromosome 14 open reading frame 166B, partial [Ornithorhynchus anatinus]
isogroup04966	PREDICTED: serine palmitoyltransferase, long chain base subunit 1 [Taeniopygia guttata]
isogroup04990	PREDICTED: similar to phospholipase A2-activating protein [Taeniopygia guttata]
isogroup05028	PREDICTED: reelin [Taeniopygia guttata]
isogroup05035	NA
isogroup05061	PREDICTED: adaptor-related protein complex 2, beta 1 subunit-like isoform 4 [Oryctolagus cuniculus]
isogroup05085	PREDICTED: hypothetical protein [Monodelphis domestica]
isogroup05098	NA
isogroup05113	NA
isogroup05219	PREDICTED: similar to eukaryotic translation initiation factor 4 gamma, 3 [Gallus gallus]
isogroup05301	PREDICTED: hypothetical protein [Gallus gallus]
isogroup05423	NA
isogroup05426	PREDICTED: similar to B-cell CLL/lymphoma 9 [Gallus gallus]
isogroup05531	NA

Isogroup	Annotation from nr Database
isogroup05713	PREDICTED: similar to nudix (nucleoside diphosphate linked moiety X)-type motif 12 [Taeniopygia guttata]
isogroup05751	PREDICTED: phospholipase C, beta 1 (phosphoinositide-specific) [Taeniopygia guttata]
isogroup05775	NA
isogroup05788	PREDICTED: ecto-NOX disulfide-thiol exchanger 2 [Taeniopygia guttata]
isogroup05797	PREDICTED: multiple PDZ domain protein [Taeniopygia guttata]
isogroup05802	PREDICTED: CD82 molecule [Taeniopygia guttata]
isogroup05906	PREDICTED: golgi membrane protein 1 [Taeniopygia guttata]
isogroup05913	PREDICTED: microtubule-associated protein 1B isoform 1 [Taeniopygia guttata]
isogroup05914	PREDICTED: microtubule-associated protein 1B isoform 1 [Taeniopygia guttata]
isogroup05930	PREDICTED: KIAA0020 [Taeniopygia guttata]
isogroup05946	NA
isogroup06021	PREDICTED: synuclein, alpha interacting protein (synphilin) [Taeniopygia guttata]
isogroup06072	PREDICTED: similar to plasma membrane calcium ATPase 2 isoform 5 [Gallus gallus]
isogroup06201	NA
isogroup06263	PREDICTED: adenomatous polyposis coli [Taeniopygia guttata]
isogroup06318	PREDICTED: RIO kinase 2 [Taeniopygia guttata]
isogroup06427	NA
isogroup06501	PREDICTED: microtubule-associated protein 1B isoform 2 [Taeniopygia guttata]
isogroup06512	NA
isogroup06584	PREDICTED: similar to transcription factor AP2-alpha isoform 2 [Ornithorhynchus anatinus]
isogroup06899	PREDICTED: serum response factor binding protein 1 [Taeniopygia guttata]
isogroup06901	PREDICTED: similar to transmembrane protein 63B [Gallus gallus]
isogroup06935	E3 ubiquitin-protein ligase BRE1A [Gallus gallus]
isogroup06963	phosphoinositide-3-kinase, class 3 [Mus musculus]
isogroup07010	PREDICTED: insulin-like growth factor 1 receptor [Taeniopygia guttata]
isogroup07071	PREDICTED: similar to MGC83004 protein [Taeniopygia guttata]
isogroup07159	putative stathmin-like 3 [Taeniopygia guttata]
isogroup07161	PREDICTED: similar to RING-finger protein [Taeniopygia guttata]
isogroup07178	NA
isogroup07263	PREDICTED: KIAA0368 [Taeniopygia guttata]
isogroup07269	NA
isogroup07298	PREDICTED: family with sequence similarity 108, member B1 [Taeniopygia guttata]

Isogroup	Annotation from nr Database
isogroup07356	PREDICTED: hypothetical protein [Taeniopygia guttata]
isogroup07379	PREDICTED: similar to protocadherin 9 [Sus scrofa]
isogroup07387	NA
isogroup07477	PREDICTED: solute carrier family 41, member 2 [Taeniopygia guttata]
isogroup07535	transcription factor 3 (E2A immunoglobulin enhancer binding factors E12/E47) [Gallus gallus]
isogroup07573	NA
isogroup07587	PREDICTED: leukemia inhibitory factor receptor [Taeniopygia guttata]
isogroup07867	PREDICTED: DEAD (Asp-Glu-Ala-Asp) box polypeptide 58 [Taeniopygia guttata]
isogroup07960	NA
isogroup08049	NA
isogroup08103	NA
isogroup08152	NA
isogroup08183	NA
isogroup08215	PREDICTED: similar to glycerophosphodiester phosphodiesterase domain containing 2 [Taeniopygia guttata]
isogroup08222	NA
isogroup08266	NA
isogroup08325	PREDICTED: YTH domain containing 2 [Oryctolagus cuniculus]
isogroup08376	PREDICTED: SH3 domain binding glutamic acid-rich protein like 2 [Taeniopygia guttata]
isogroup08418	PREDICTED: similar to TBC1 domain family member 2A [Taeniopygia guttata]
isogroup08425	PREDICTED: similar to KIAA1438 protein [Gallus gallus]
isogroup08448	PREDICTED: IQ motif containing E [Taeniopygia guttata]
isogroup08450	PREDICTED: hypothetical protein, partial [Gallus gallus]
isogroup08473	PREDICTED: similar to KIAA0700 protein [Taeniopygia guttata]
isogroup08490	NA
isogroup08617	NA
isogroup08621	NA
isogroup08663	NA
isogroup08825	NA
isogroup08834	PREDICTED: alpha-L-iduronidase [Taeniopygia guttata]
isogroup08873	NA
isogroup08884	NA
isogroup08899	NA
isogroup08971	NA

Isogroup	Annotation from nr Database
isogroup09101	PREDICTED: similar to protein phosphatase 1F (PP2C domain containing) [Taeniopygia guttata]
isogroup09192	NA
isogroup09364	unnamed protein product [Mus musculus]
isogroup09588	NA
isogroup09612	PREDICTED: similar to yeast ribosomal protein S28 homologue [Gallus gallus]
isogroup09646	PREDICTED: hypothetical protein [Gallus gallus]
isogroup09745	NA
isogroup09920	NA
isogroup10025	PREDICTED: similar to family with sequence similarity 102, member A [Taeniopygia guttata]
isogroup10103	PREDICTED: hypothetical protein [Taeniopygia guttata]
isogroup10113	PREDICTED: cytoglobin [Taeniopygia guttata]
isogroup10114	PREDICTED: cytoglobin [Taeniopygia guttata]
isogroup10177	PREDICTED: monoglyceride lipase [Taeniopygia guttata]
isogroup10195	PREDICTED: similar to BTB (POZ) domain containing 4 [Canis familiaris]
isogroup10211	NA
isogroup10438	PREDICTED: hypothetical protein [Taeniopygia guttata]
isogroup10487	PREDICTED: LIM domain kinase 2 [Taeniopygia guttata]
isogroup10565	PREDICTED: myeloid/lymphoid or mixed-lineage leukemia (trithorax homolog, Drosophila) [Taeniopygia guttata]
isogroup10596	NA
isogroup10628	PREDICTED: inositol 1,4,5-triphosphate receptor-I isoform 4 [Oryctolagus cuniculus]
isogroup10646	NA
isogroup10647	NA
isogroup10656	NA
isogroup10668	PREDICTED: p21 protein (Cdc42/Rac)-activated kinase 3 isoform 1 [Taeniopygia guttata]
isogroup10717	NA
isogroup10743	PREDICTED: patched [Taeniopygia guttata]
isogroup10773	PREDICTED: hypothetical protein [Taeniopygia guttata]
isogroup10827	PREDICTED: similar to formin 2 [Taeniopygia guttata]
isogroup11007	PREDICTED: pleckstrin and Sec7 domain containing 3 [Taeniopygia guttata]
isogroup11082	myelin proteolipid protein [Bos taurus]
isogroup11083	PREDICTED: similar to hydroxysteroid dehydrogenase like 2 [Taeniopygia guttata]
isogroup11134	NA

Isogroup	Annotation from nr Database
isogroup11150	PREDICTED: similar to KIAA2026 [Pan troglodytes]
isogroup11155	PREDICTED: solute carrier family 45, member 1 [Taeniopygia guttata]
isogroup11177	unnamed protein product [Homo sapiens]
isogroup11231	PREDICTED: similar to KIAA0372 [Gallus gallus]
isogroup11238	PREDICTED: protein inhibitor of activated STAT X [Taeniopygia guttata]
isogroup11420	PREDICTED: putative clathrin light polypeptide variant 1a isoform 2 [Taeniopygia guttata]
isogroup11472	PREDICTED: similar to Mediator of RNA polymerase II transcription subunit 1 [Taeniopygia guttata]
isogroup11512	NA
isogroup11520	PREDICTED: hypothetical protein [Taeniopygia guttata]
isogroup11674	PREDICTED: similar to cAMP responsive element binding protein 3-like 3 [Taeniopygia guttata]
isogroup11881	PREDICTED: hypothetical protein [Taeniopygia guttata]
isogroup12006	PREDICTED: hypothetical protein [Gallus gallus]
isogroup12018	NA
isogroup12023	NA
isogroup12033	PREDICTED: similar to phosphodiesterase 8B [Taeniopygia guttata]
isogroup12077	thioredoxin 1 [Melopsittacus undulatus]
isogroup12138	PREDICTED: similar to KIAA0833 protein [Taeniopygia guttata]
isogroup12163	PREDICTED: integrator complex subunit 4 [Taeniopygia guttata]
isogroup12182	NA
isogroup12194	PREDICTED: KIAA0258 [Taeniopygia guttata]
isogroup12217	NA
isogroup12256	PREDICTED: inhibitor of kappa light polypeptide gene enhancer in B-cells, kinase complex-associated protein [Taeniopygia guttata]
isogroup12315	NA
isogroup12327	PREDICTED: similar to rapamycin-insensitive companion of mTOR [Taeniopygia guttata]
isogroup12340	PREDICTED: Kv channel interacting protein 2 [Taeniopygia guttata]
isogroup12421	NA
isogroup12530	carboxypeptidase D [Anas platyrhynchos]
isogroup12583	PREDICTED: putative dynactin light chain 3 [Taeniopygia guttata]
isogroup12585	NA
isogroup12666	PREDICTED: similar to chorea-acanthocytosis [Taeniopygia guttata]
isogroup12760	PREDICTED: WD repeat domain 36 [Taeniopygia guttata]
isogroup12855	PREDICTED: similar to NDUFA12-like [Gallus gallus]

Isogroup	Annotation from nr Database
isogroup12913	PREDICTED: similar to peptidylprolyl isomerase domain and WD repeat containing 1 [Taeniopygia guttata]
isogroup12934	PREDICTED: fem-1 homolog c (C. elegans) [Taeniopygia guttata]
isogroup13022	NA
isogroup13032	NA
isogroup13045	NA
isogroup13098	PREDICTED: NF-M [Taeniopygia guttata]
isogroup13101	PREDICTED: similar to phosphatidylinositol glycan, class G [Taeniopygia guttata]
isogroup13110	NA
isogroup13125	PREDICTED: similar to cyclin G associated kinase [Taeniopygia guttata]
isogroup13137	PREDICTED: similar to ankyrin repeat domain protein 17 [Gallus gallus]
isogroup13220	NA
isogroup13247	NA
isogroup13255	PREDICTED: similar to diacylglycerol kinase, theta [Taeniopygia guttata]
isogroup13285	PREDICTED: adenomatous polyposis coli [Taeniopygia guttata]
isogroup13304	PREDICTED: similar to DIRAS family protein [Taeniopygia guttata]
isogroup13357	PREDICTED: SMC5 protein [Taeniopygia guttata]
isogroup13388	PREDICTED: hypothetical protein [Taeniopygia guttata]
isogroup13392	PREDICTED: similar to RUN and SH3 domain containing 2 [Taeniopygia guttata]
isogroup13455	NA
isogroup13499	PREDICTED: neurolysin (metallopeptidase M3 family) [Taeniopygia guttata]
isogroup13518	NA
isogroup13712	PREDICTED: small glutamine-rich tetratricopeptide repeat (TPR)-containing, beta [Taeniopygia guttata]
isogroup13741	NA
isogroup13761	PREDICTED: putative Adenylate kinase 3 alpha like 1 variant 1 [Taeniopygia guttata]
isogroup13887	PREDICTED: superkiller viralicidic activity 2-like 2 [Taeniopygia guttata]
isogroup13892	PREDICTED: fucosyltransferase 10 (alpha (1,3) fucosyltransferase) [Taeniopygia guttata]
isogroup13911	NA
isogroup14031	carboxypeptidase D [Anas platyrhynchos]
isogroup14246	chromo-helicase DNA-binding protein [Zonotrichia albicollis]
isogroup14285	PREDICTED: WD repeat domain 36 [Taeniopygia guttata]
isogroup14475	PREDICTED: similar to 2210408I21Rik protein [Taeniopygia guttata]
isogroup14499	PREDICTED: hypothetical protein [Taeniopygia guttata]

Isogroup	Annotation from nr Database
isogroup14583	PREDICTED: platelet-activating factor acetylhydrolase, isoform Ib, beta subunit 30kDa [Taeniopygia guttata]
isogroup14614	PRMT1 protein [Homo sapiens]
isogroup14692	PREDICTED: similar to ribosomal protein L7-like 1 [Taeniopygia guttata]
isogroup14706	PREDICTED: inhibitor of kappa light polypeptide gene enhancer in B-cells, kinase complex-associated protein [Taeniopygia guttata]
isogroup14719	PREDICTED: similar to Katanin p60 ATPase-containing subunit A-like 2 [Taeniopygia guttata]
isogroup14749	PREDICTED: chromosome 9 open reading frame 95 [Taeniopygia guttata]
isogroup14750	PREDICTED: hypothetical protein [Taeniopygia guttata]
isogroup14767	PREDICTED: similar to Centrin, EF-hand protein, 3 (CDC31 homolog, yeast) [Gallus gallus]
isogroup14784	NA
isogroup14801	NA
isogroup14823	PREDICTED: similar to chorea-acanthocytosis [Taeniopygia guttata]
isogroup14880	PREDICTED: ankyrin repeat, family A (RFXANK-like), 2 [Taeniopygia guttata]
isogroup14905	PREDICTED: excision repair cross-complementing rodent repair deficiency, complementation group 8 [Taeniopygia guttata]
isogroup15050	PREDICTED: MAP-kinase activating death domain isoform 2 [Taeniopygia guttata]
isogroup15067	PREDICTED: spectrin, alpha, non-erythrocytic 1 (alpha-fodrin) isoform 1 [Taeniopygia guttata]
isogroup15123	putative autophagy 12-like [Taeniopygia guttata]
isogroup15166	PREDICTED: hypothetical protein [Taeniopygia guttata]
isogroup15221	NA
isogroup15225	PREDICTED: haloacid dehalogenase-like hydrolase domain containing 2 [Taeniopygia guttata]
isogroup15228	PREDICTED: phospholipase C, beta 1 (phosphoinositide-specific) [Taeniopygia guttata]
isogroup15289	PREDICTED: KIAA0368 [Taeniopygia guttata]
isogroup15296	PREDICTED: similar to semaphorin SEMA6A1 [Gallus gallus]
isogroup15384	methylcrotonoyl-Coenzyme A carboxylase 2 (beta) [Xenopus laevis]
isogroup15457	NA
isogroup15468	PREDICTED: protocadherin 9 [Taeniopygia guttata]
isogroup15511	NA
isogroup15537	NA
isogroup15645	NA
isogroup15674	sodium/potassium-transporting ATPase subunit alpha-3 [Gallus gallus]
isogroup15684	PREDICTED: similar to solute carrier family 1 (neuronal/epithelial high affinity glutamate transporter, system Xag), member 1 [Taeniopygia guttata]

Isogroup	Annotation from nr Database
isogroup15700	PREDICTED: similar to RING-finger protein [Taeniopygia guttata]
isogroup15718	NA
isogroup15800	PREDICTED: hypothetical protein [Gallus gallus]
isogroup15822	PREDICTED: pentatricopeptide repeat domain 2 [Taeniopygia guttata]
isogroup15855	NA
isogroup15857	NA
isogroup15869	PREDICTED: RMI1, RecQ mediated genome instability 1, homolog [Taeniopygia guttata]
isogroup15889	protein mab-21-like 1 [Homo sapiens]
isogroup15905	PREDICTED: tetratricopeptide repeat domain 33 [Taeniopygia guttata]
isogroup15918	PREDICTED: corneal wound healing-related protein [Taeniopygia guttata]
isogroup15928	PREDICTED: similar to DnaJ (Hsp40) homolog, subfamily C, member 21 [Taeniopygia guttata]
isogroup16017	PREDICTED: hypothetical protein [Taeniopygia guttata]
isogroup16030	NA
isogroup16139	PREDICTED: similar to KIAA1281 protein [Taeniopygia guttata]
isogroup16198	NA
isogroup16213	PREDICTED: corneal wound healing-related protein [Taeniopygia guttata]
isogroup16268	PREDICTED: centrosomal protein 78kDa [Taeniopygia guttata]
isogroup16272	NA
isogroup16303	PREDICTED: similar to Hepatocyte nuclear factor 6 (HNF-6) (One cut domain family member 1) isoform 1 [Canis familiaris]
isogroup16345	hydroxysteroid (17-beta) dehydrogenase 4 [Gallus gallus]
isogroup16360	PREDICTED: aprataxin [Taeniopygia guttata]
isogroup16435	NA
isogroup16459	NA
isogroup16535	NA
isogroup16556	PREDICTED: SEC24 family, member C (S. cerevisiae) [Taeniopygia guttata]
isogroup16568	PREDICTED: neurotrophic tyrosine kinase, receptor, type 2, partial [Taeniopygia guttata]
isogroup16569	AF355752_1 reverse transcriptase [Chelonia mydas]
isogroup16607	PREDICTED: hyperpolarization activated cyclic nucleotide-gated potassium channel 2 [Taeniopygia guttata]
isogroup16608	NA
isogroup16622	NA
isogroup16656	PREDICTED: general transcription factor IIH, polypeptide 2, 44kD subunit isoform 8 [Pan troglodytes]
isogroup16713	NA

Isogroup	Annotation from nr Database
isogroup16722	NA
isogroup16723	PREDICTED: LMBR1 domain containing 2 [Taeniopygia guttata]
isogroup16831	NA
isogroup16879	PREDICTED: similar to Small nuclear RNA activating complex, polypeptide 3 [Gallus gallus]
isogroup16885	NA
isogroup16937	PREDICTED: inositol 1,3,4-triphosphate 5/6 kinase [Taeniopygia guttata]
isogroup16964	NA
isogroup17042	NA
isogroup17083	PREDICTED: similar to AP-3 complex subunit beta-1 [Taeniopygia guttata]
isogroup17092	PREDICTED: similar to protein tyrosine kinase fer isoform 1 [Bos taurus]
isogroup17106	PREDICTED: similar to alpha-L-iduronidase, partial [Taeniopygia guttata]
isogroup17110	NA
isogroup17131	NA
isogroup17154	PREDICTED: minichromosome maintenance complex component 3 associated protein [Taeniopygia guttata]
isogroup17195	PREDICTED: hypothetical protein [Taeniopygia guttata]
isogroup17198	PREDICTED: SET binding protein 1 [Taeniopygia guttata]
isogroup17210	PREDICTED: hypothetical protein [Taeniopygia guttata]
isogroup17243	PREDICTED: transient receptor potential cation channel, subfamily M, member 3 [Taeniopygia guttata]
isogroup17286	NA
isogroup17296	PREDICTED: histidine acid phosphatase domain containing 1 [Taeniopygia guttata]
isogroup17309	PREDICTED: upstream binding transcription factor, RNA polymerase I [Equus caballus]
isogroup17356	PREDICTED: similar to ring finger protein 38 [Monodelphis domestica]
isogroup17372	talin 1 [Gallus gallus]
isogroup17408	PREDICTED: polymerase (DNA directed) kappa, partial [Taeniopygia guttata]
isogroup17573	PREDICTED: sema domain, immunoglobulin domain (Ig), transmembrane domain (TM) and short cytoplasmic domain, (semaphorin) 4D [Taeniopygia guttata]
isogroup17579	NA
isogroup17604	sorting nexin 2 [Gallus gallus]
isogroup17609	NA
isogroup17874	PREDICTED: hypothetical protein [Taeniopygia guttata]
isogroup17888	NA
isogroup17919	PREDICTED: calsyntenin 1 [Taeniopygia guttata]
isogroup17971	NA

**Isogroup**	**Annotation from nr Database**
isogroup18008	PREDICTED: protein inhibitor of activated STAT X [Taeniopygia guttata]
isogroup18135	PREDICTED: hypothetical protein [Taeniopygia guttata]
isogroup18252	PREDICTED: similar to cytoplasmic dynein 1 heavy chain 1 [Taeniopygia guttata]
isogroup18497	putative DnaJ subfamily A member 1 variant 3 [Taeniopygia guttata]
isogroup18615	kinesin family member 3B [Gallus gallus]
isogroup18645	NA
isogroup18708	PREDICTED: similar to Hippocampus abundant transcript-like protein 1 [Taeniopygia guttata]
isogroup18739	PREDICTED: hypothetical protein [Taeniopygia guttata]
isogroup18743	PREDICTED: similar to G elongation factor, mitochondrial 2 [Taeniopygia guttata]
isogroup18777	PREDICTED: insulin-like growth factor 1 receptor [Taeniopygia guttata]
isogroup18865	NA
isogroup18887	PREDICTED: similar to ring finger protein 38 [Monodelphis domestica]
isogroup18904	NA
isogroup19029	PREDICTED: fer (fps/fes related) tyrosine kinase (phosphoprotein NCP94) [Taeniopygia guttata]
isogroup19076	PREDICTED: similar to KIAA1119 protein [Monodelphis domestica]
isogroup19160	PREDICTED: solute carrier family 12 (sodium/potassium/chloride transporters), member 2 [Taeniopygia guttata]
isogroup19188	NA
isogroup19270	NA
isogroup19414	NA
isogroup19416	PREDICTED: neuronal protein 3.1 [Taeniopygia guttata]
isogroup19477	PREDICTED: hypothetical protein [Taeniopygia guttata]
isogroup19565	NA
isogroup19575	NA
isogroup19619	NA
isogroup19650	NA
isogroup19721	guanine nucleotide-binding protein G(I)/G(S)/G(O) subunit gamma-3 precursor [Mus musculus]
isogroup19808	PREDICTED: fibroblast growth factor 10 [Taeniopygia guttata]
isogroup20096	NA
isogroup20144	NA
isogroup20211	PREDICTED: similar to arylsulfatase B [Taeniopygia guttata]
isogroup20235	PREDICTED: signal-induced proliferation-associated 1 like 2 [Taeniopygia guttata]
isogroup20237	PREDICTED: centrosomal protein 78kDa [Taeniopygia guttata]
isogroup20295	NA

Isogroup	Annotation from nr Database
isogroup20328	PREDICTED: hypothetical protein [Taeniopygia guttata]
isogroup20463	PREDICTED: similar to cyclin G associated kinase [Taeniopygia guttata]
isogroup20470	PREDICTED: similar to DnaJ (Hsp40) homolog, subfamily C, member 21 [Taeniopygia guttata]
isogroup20549	NA
isogroup20555	PREDICTED: similar to transcription factor IIIB 150 [Taeniopygia guttata]
isogroup20562	NA
isogroup20569	PREDICTED: similar to deleted in liver cancer 1 [Taeniopygia guttata]
isogroup20578	NA
isogroup20683	NA
isogroup20772	PREDICTED: hypothetical protein [Taeniopygia guttata]
isogroup20776	PREDICTED: similar to TBC1 domain family member 2A [Taeniopygia guttata]
isogroup20800	NA
isogroup20829	NA
isogroup20938	NA
isogroup20993	PREDICTED: similar to oxytocinase splice [Gallus gallus]
isogroup21006	PREDICTED: cyclin-dependent kinase-like 5 [Taeniopygia guttata]
isogroup21015	NA
isogroup21055	NA
isogroup21092	PREDICTED: similar to KIAA0456 protein [Taeniopygia guttata]
isogroup21194	PREDICTED: amyloid beta (A4) precursor protein-binding, family A, member 1 [Taeniopygia guttata]
isogroup21279	PREDICTED: jumonji domain containing 2C [Taeniopygia guttata]
isogroup21281	PREDICTED: hypothetical protein [Taeniopygia guttata]
isogroup21321	PREDICTED: similar to monogenic, audiogenic seizure susceptibility 1 homolog (mouse), [Monodelphis domestica]
isogroup21329	PREDICTED: WD repeat domain 41 [Taeniopygia guttata]
isogroup21350	NA
isogroup21399	NA
isogroup21426	PREDICTED: endothelial differentiation, lysophosphatidic acid G-protein-coupled receptor, 2 [Taeniopygia guttata]
isogroup21436	PREDICTED: similar to PLCG1 variant protein [Monodelphis domestica]
isogroup21454	PREDICTED: interleukin 6 signal transducer (gp130, oncostatin M receptor) [Taeniopygia guttata]
isogroup21606	NA
isogroup21656	PREDICTED: similar to ubiquitin-like with PHD and ring finger domains 2, partial [Taeniopygia guttata]
isogroup21778	PREDICTED: similar to C1orf15 [Gallus gallus]

Isogroup	Annotation from nr Database
isogroup21857	PREDICTED: similar to phosphatidylinositol glycan, class G [Taeniopygia guttata]
isogroup21886	PREDICTED: osteoclast stimulating factor 1 [Taeniopygia guttata]
isogroup21953	NA
isogroup22026	NA
isogroup22027	NA
isogroup22037	NA
isogroup22044	PREDICTED: hypothetical protein [Taeniopygia guttata]
isogroup22075	PREDICTED: similar to FCH domain only protein 2 [Taeniopygia guttata]
isogroup22087	hypothetical protein [Taeniopygia guttata]
isogroup22190	PREDICTED: similar to ankyrin repeat-containing cofactor-1 [Gallus gallus]
isogroup22217	NA
isogroup22257	PREDICTED: hypothetical protein [Taeniopygia guttata]
isogroup22262	PREDICTED: zinc finger, CCHC domain containing 6 [Taeniopygia guttata]
isogroup22270	PREDICTED: similar to guanine nucleotide exchange factor [Gallus gallus]
isogroup22357	NA
isogroup22417	PREDICTED: hypothetical protein [Taeniopygia guttata]
isogroup22481	PREDICTED: hypothetical protein [Gallus gallus]
isogroup22504	PREDICTED: similar to dishevelled-associated activator of morphogenesis 1 [Gallus gallus]
isogroup22507	NA
isogroup22548	coatomer protein complex, subunit alpha [Gallus gallus]
isogroup22565	PREDICTED: microtubule-associated protein 1A [Taeniopygia guttata]
isogroup22576	PREDICTED: hypothetical protein [Gallus gallus]
isogroup22601	PREDICTED: similar to X-ray repair cross complementing protein 4 [Taeniopygia guttata]
isogroup22667	NA
isogroup22700	PREDICTED: similar to axonal transport of synaptic vesicles isoform 2 [Ornithorhynchus anatinus]
isogroup22706	NA
isogroup22714	NA
isogroup22726	PREDICTED: multiple PDZ domain protein [Taeniopygia guttata]
contig90135	NA
contig90188	NA

Supplementary Table 2. Genes expressed higher in females than males in the medial amygdala.

Isogroup	Annotation from nr Database
isogroup00008	PREDICTED: similar to UTP18, small subunit processome component [Taeniopygia guttata]
isogroup00027	PREDICTED: similar to coiled-coil domain containing 5 [Taeniopygia guttata]
isogroup00028	adipocyte differentiation-related protein [Gallus gallus]
isogroup00032	NA
isogroup00052	NA
isogroup00073	NA
isogroup00097	PREDICTED: transforming, acidic coiled-coil containing protein 3 [Taeniopygia guttata]
isogroup00147	NA
isogroup00182	PREDICTED: similar to pol-like protein ENS-3 [Taeniopygia guttata]
isogroup00204	PREDICTED: hypothetical protein [Gallus gallus]
isogroup00230	NA
isogroup00260	PREDICTED: similar to Gag-Pro-Pol-Env protein [Taeniopygia guttata]
isogroup00396	PREDICTED: similar to KIAA1892 protein - DDB1- and CUL4-associated factor 12 [Monodelphis domestica]
isogroup00459	NA
isogroup00499	PREDICTED: hypothetical protein [Taeniopygia guttata]
isogroup00520	PREDICTED: metallophosphoesterase 1 [Taeniopygia guttata]
isogroup00545	PREDICTED: hypothetical protein [Taeniopygia guttata]
isogroup00584	PREDICTED: phosphotriesterase related [Taeniopygia guttata]
isogroup00657	PREDICTED: similar to POLG2 protein [Taeniopygia guttata]
isogroup00660	PREDICTED: similar to polyprotein [Taeniopygia guttata]
isogroup00829	PREDICTED: collagen, type IV, alpha 3 (Goodpasture antigen) binding protein [Taeniopygia guttata]
isogroup00842	PREDICTED: similar to Gag-Pro-Pol-Env protein [Taeniopygia guttata]
isogroup00849	activated RNA polymerase II transcriptional coactivator p15 [Gallus gallus]
isogroup00993	PREDICTED: similar to interleukin 22-binding protein [Taeniopygia guttata]
isogroup01113	NA
isogroup01222	PREDICTED: similar to pol-like protein ENS-3, partial [Taeniopygia guttata]
isogroup01269	valosin-containing protein [Gallus gallus]
isogroup01578	NA
isogroup01716	PREDICTED: similar to potassium channel modulatory factor 1 [Taeniopygia guttata]
isogroup01999	NA
isogroup02339	PREDICTED: similar to opioid receptor, kappa 1 [Taeniopygia guttata]
isogroup02469	putative protein kinase C inhibitor/ASWZ [Taeniopygia guttata]

Isogroup	Annotation from nr Database
isogroup02829	PREDICTED: similar to ARKadia-like 1 isoform 2 [Taeniopygia guttata]
isogroup02863	NA
isogroup02965	PREDICTED: similar to WD repeat domain 51A [Gallus gallus]
isogroup03020	PREDICTED: ankyrin repeat, SAM and basic leucine zipper domain containing 1 [Taeniopygia guttata]
isogroup03130	NA
isogroup03190	PREDICTED: hypothetical protein [Gallus gallus]
isogroup03286	PREDICTED: similar to early response to neural induction ERNI [Gallus gallus]
isogroup03670	NA
isogroup03755	putative neurotensin variant 1 [Taeniopygia guttata]
isogroup03875	PREDICTED: hypothetical protein [Gallus gallus]
isogroup03917	NA
isogroup04099	NA
isogroup04108	PREDICTED: centromere protein Q [Taeniopygia guttata]
isogroup04353	NA
isogroup04474	NA
isogroup04509	PREDICTED: similar to Adenylate kinase isoenzyme 5, partial [Taeniopygia guttata]
isogroup04679	NA
isogroup04702	NA
isogroup04710	unnamed protein product [Homo sapiens]
isogroup04876	NA
isogroup04878	NA
isogroup04887	PREDICTED: enoyl Coenzyme A hydratase domain containing 2 [Taeniopygia guttata]
isogroup04912	NA
isogroup05078	NA
isogroup05190	PREDICTED: similar to Gag polyprotein [Taeniopygia guttata]
isogroup05470	NA
isogroup05551	NA
isogroup05793	PREDICTED: similar to polyprotein [Taeniopygia guttata]
isogroup05794	NA
isogroup06101	PREDICTED: similar to rCG43799 [Taeniopygia guttata]
isogroup06103	NA
isogroup06151	NA
isogroup06322	NA
isogroup06326	tumor necrosis factor, alpha-induced protein 6 [Gallus gallus]

Isogroup	Annotation from nr Database
isogroup06361	NA
isogroup06396	NA
isogroup06400	YK047_HUMAN RecName: Full=Putative Ig-like domain-containing protein DKFZp686O24166/DKFZp686I21167; Flags: Precursor
isogroup06423	NA
isogroup06470	PREDICTED: similar to Gag-Pro-Pol-Env protein [Taeniopygia guttata]
isogroup06762	NA
isogroup06853	NA
isogroup07047	NA
isogroup07054	PREDICTED: hypothetical protein [Taeniopygia guttata]
isogroup07155	NA
isogroup07236	NA
isogroup07917	NA
isogroup07950	MC4R [Anser anser]
isogroup08029	NA
isogroup08046	NA
isogroup08283	NA
isogroup08540	NA
isogroup08616	NA
isogroup08914	NA
isogroup09039	NA
isogroup09204	NA
isogroup09649	PREDICTED: hypothetical protein [Taeniopygia guttata]
isogroup09947	NA
isogroup10000	NA
isogroup10287	NA
isogroup10489	NA
isogroup10497	NA
isogroup10572	hypothetical protein LOC422926 [Gallus gallus]
isogroup10667	transcription factor BTF3 isoform 1 [Mus musculus]
isogroup10762	NA
isogroup10781	PREDICTED: similar to Gag-Pro-Pol protein [Taeniopygia guttata]
isogroup10805	NA
isogroup10971	NA
isogroup11168	PREDICTED: similar to gag-pro-pol polyprotein [Taeniopygia guttata]
isogroup11389	NA

Isogroup	Annotation from nr Database
isogroup11430	NA
isogroup11507	NA
isogroup11565	NA
isogroup12398	NA
isogroup12400	PREDICTED: similar to polymerase [Taeniopygia guttata]
isogroup12731	NA
isogroup12784	PREDICTED: retinal G-protein coupled receptor [Taeniopygia guttata]
isogroup13068	PREDICTED: similar to MEX3C [Ornithorhynchus anatinus]
isogroup13176	NA
isogroup13527	NA
isogroup13857	PREDICTED: hypothetical protein [Gallus gallus]
isogroup13902	NA
isogroup14050	PREDICTED: putative preproenkephalin 1 [Taeniopygia guttata]
isogroup14064	PREDICTED: similar to pol-like protein ENS-3, partial [Taeniopygia guttata]
isogroup14780	NA
isogroup14976	NA
isogroup15028	NA
isogroup15127	hypothetical protein PANDA_012261 [Ailuropoda melanoleuca]
isogroup15585	NA
isogroup15617	ribosomal protein S13 [synthetic construct]
isogroup15653	PREDICTED: similar to pol protein [Ornithorhynchus anatinus]
isogroup15823	PREDICTED: galanin receptor 3 [Taeniopygia guttata]
isogroup16070	NA
isogroup16168	PREDICTED: hypothetical protein [Taeniopygia guttata]
isogroup16200	PREDICTED: similar to Envelope glycoprotein [Taeniopygia guttata]
isogroup16214	PREDICTED: similar to trans-Golgi protein GMx33 [Gallus gallus]
isogroup16245	NA
isogroup16354	PREDICTED: similar to Gag-Pro-Pol-Env protein [Taeniopygia guttata]
isogroup16521	NA
isogroup16621	NA
isogroup16842	PREDICTED: similar to mCG21813 [Taeniopygia guttata]
isogroup16889	PREDICTED: similar to polymerase (RNA) III (DNA directed) polypeptide H (22.9kD) isoform 2 [Taeniopygia guttata]
isogroup17167	NA
isogroup17181	NA
isogroup17199	POL-like [Gallus gallus]

Isogroup	Annotation from nr Database
isogroup17235	NA
isogroup17458	PREDICTED: zinc finger, CCHC domain containing 4 [Taeniopygia guttata]
isogroup17762	NA
isogroup18340	NA
isogroup18344	PREDICTED: PDZ domain containing RING finger 4 [Taeniopygia guttata]
isogroup18919	NA
isogroup18978	PREDICTED: similar to polymerase [Taeniopygia guttata]
isogroup19033	NA
isogroup19043	env [Bonasa umbellus]
isogroup19260	NA
isogroup19287	PREDICTED: TATA box binding protein (TBP)-associated factor, RNA polymerase I, B, 63kDa [Taeniopygia guttata]
isogroup19308	PREDICTED: solute carrier family 18 (vesicular monoamine), member 2 [Taeniopygia guttata]
isogroup19373	hypothetical protein [Gallus gallus]
isogroup19448	PREDICTED: similar to pol protein [Taeniopygia guttata]
isogroup19487	NA
isogroup19506	NA
isogroup19515	NA
isogroup19542	NA
isogroup19731	NA
isogroup19892	cdc48 [Larimichthys crocea]
isogroup20100	PREDICTED: similar to alpha-kinase 2 [Taeniopygia guttata]
isogroup20441	NA
isogroup20545	NA
isogroup20788	NA
isogroup21027	PREDICTED: hypothetical protein [Taeniopygia guttata]
isogroup21227	NA
isogroup21286	NA
isogroup21319	NA
isogroup21653	NA
isogroup21676	NA
isogroup21763	PREDICTED: similar to Gag-Pro-Pol-Env protein [Taeniopygia guttata]
isogroup21866	NA
isogroup22080	NA
isogroup22154	NA

Isogroup	Annotation from nr Database
isogroup22206	mKIAA1376 protein [Mus musculus]
isogroup22521	NA
isogroup22599	PREDICTED: v-Ha-ras Harvey rat sarcoma viral oncogene homolog [Taeniopygia guttata]
isogroup22629	NA
contig90185	NA

Supplementary Table 3. Gene Ontology terms over-represented among genes significantly differentially expressed between males and females in the medial amygdala.

GO ID	GO Description
GO:0000079	regulation of cyclin-dependent protein kinase activity
GO:0000910	cytokinesis
GO:0001649	osteoblast differentiation
GO:0006916	anti-apoptosis
GO:0007018	microtubule-based movement
GO:0007019	microtubule depolymerization
GO:0007033	vacuole organization
GO:0007265	Ras protein signal transduction
GO:0009636	response to toxin
GO:0010740	positive regulation of intracellular protein kinase cascade
GO:0014075	response to amine stimulus
GO:0019058	viral infectious cycle
GO:0030041	actin filament polymerization
GO:0030307	positive regulation of cell growth
GO:0031016	pancreas development
GO:0031103	axon regeneration
GO:0031269	pseudopodium assembly
GO:0031929	TOR signaling cascade
GO:0033273	response to vitamin
GO:0034968	histone lysine methylation
GO:0043627	response to estrogen stimulus
GO:0045841	negative regulation of mitotic metaphase/anaphase transition
GO:0046519	sphingoid metabolic process
GO:0048732	gland development
GO:0050673	epithelial cell proliferation
GO:0051313	attachment of spindle microtubules to chromosome
GO:0051914	positive regulation of synaptic plasticity by chemical substance
GO:0060429	epithelium development
GO:0070507	regulation of microtubule cytoskeleton organization
GO:0004620	phospholipase activity
GO:0004713	protein tyrosine kinase activity
GO:0004896	cytokine receptor activity
GO:0005496	steroid binding
GO:0008528	G-protein coupled peptide receptor activity

GO ID	GO Description
GO:0015631	tubulin binding
GO:0016408	C-acyltransferase activity
GO:0019207	kinase regulator activity
GO:0019838	growth factor binding
GO:0019900	kinase binding
GO:0032559	adenyl ribonucleotide binding
GO:0035004	phosphatidylinositol 3-kinase activity
GO:0050839	cell adhesion molecule binding
GO:0015630	microtubule cytoskeleton
GO:0043005	neuron projection
GO:0044430	cytoskeletal part
GO:0044431	Golgi apparatus part

Supplementary Table 4. Genes expressed higher in males than females in the hypothalamus.

Isogroup	Annotation from nr Database
isogroup00069	PREDICTED: ubiquitin associated protein 2 [Taeniopygia guttata]
isogroup00091	PREDICTED: Janus kinase 2 [Taeniopygia guttata]
isogroup00175	PREDICTED: similar to Mad2 gene product [Taeniopygia guttata]
isogroup00187	PREDICTED: similar to RNF170 protein [Taeniopygia guttata]
isogroup00224	PREDICTED: chromosome 9 open reading frame 3 [Taeniopygia guttata]
isogroup00274	putative molybdopterin synthase large subunit MOCS2B [Taeniopygia guttata]
isogroup00276	PREDICTED: centromere protein K [Taeniopygia guttata]
isogroup00365	PREDICTED: similar to DKFZP459P083 protein isoform 1 [Taeniopygia guttata]
isogroup00451	PREDICTED: similar to GRAM domain containing 3 [Taeniopygia guttata]
isogroup00458	SWI/SNF related, matrix associated, actin dependent regulator of chromatin, subfamily a, member 2 [Gallus gallus]
isogroup00524	PREDICTED: hypothetical protein [Taeniopygia guttata]
isogroup00533	DNA-directed DNA polymerase kappa [Gallus gallus]
isogroup00551	RPTOR independent companion of MTOR, complex 2 [Bos taurus]
isogroup00570	single-stranded DNA binding protein 2 [Homo sapiens]
isogroup00600	heterogeneous nuclear ribonucleoprotein K [Gallus gallus]
isogroup00663	PREDICTED: similar to phosphoinositide-3-kinase, regulatory subunit 1 (alpha) [Taeniopygia guttata]
isogroup00685	PREDICTED: ATP synthase, H+ transporting, mitochondrial F1 complex, alpha subunit [Taeniopygia guttata]
isogroup00694	PREDICTED: prolactin receptor [Taeniopygia guttata]
isogroup00700	NA
isogroup00744	PREDICTED: xeroderma pigmentosum, complementation group A [Taeniopygia guttata]
isogroup00779	PREDICTED: SMC5 protein [Taeniopygia guttata]
isogroup00783	PREDICTED: death-associated protein kinase 1 [Taeniopygia guttata]
isogroup00784	PREDICTED: similar to phosphodiesterase 1A, calmodulin-dependent [Taeniopygia guttata]
isogroup00794	PREDICTED: similar to Lipid phosphate phosphohydrolase 1 [Taeniopygia guttata]
isogroup00984	PREDICTED: similar to KIAA0372 [Gallus gallus]
isogroup00991	PREDICTED: jumonji domain containing 2C [Taeniopygia guttata]
isogroup00999	PREDICTED: COBW domain containing protein1 [Taeniopygia guttata]
isogroup01050	PREDICTED: zinc finger, CCHC domain containing 6 [Taeniopygia guttata]
isogroup01106	PREDICTED: similar to SHC (Src homology 2 domain containing) transforming protein 3 [Taeniopygia guttata]
isogroup01156	PREDICTED: similar to E-type ATPase [Gallus gallus]
isogroup01180	PREDICTED: myosin, light chain kinase [Taeniopygia guttata]

Isogroup	Annotation from nr Database
isogroup01181	PREDICTED: similar to phosphoinositide-3-kinase, class 3 [Taeniopygia guttata]
isogroup01284	PREDICTED: RAS p21 protein activator (GTPase activating protein) 1 [Taeniopygia guttata]
isogroup01427	PREDICTED: microtubule-associated protein 1B isoform 1 [Taeniopygia guttata]
isogroup01442	PREDICTED: peptidylglycine alpha-amidating monooxygenase isoform 2 [Taeniopygia guttata]
isogroup01466	PREDICTED: similar to protein geranylgeranyltransferase type I, beta subunit isoform 1 [Equus caballus]
isogroup01479	PREDICTED: similar to hook homolog 3 [Taeniopygia guttata]
isogroup01587	PREDICTED: G protein-coupled receptor 123 [Taeniopygia guttata]
isogroup01601	PREDICTED: metaxin 3 [Taeniopygia guttata]
isogroup01658	Rpl17 protein [Mus musculus]
isogroup01708	PREDICTED: similar to phosphatidylinositol transfer protein, cytoplasmic 1 [Taeniopygia guttata]
isogroup01750	PREDICTED: hypothetical protein [Taeniopygia guttata]
isogroup01786	ankyrin repeat domain 55 [Xenopus (Silurana) tropicalis]
isogroup01797	PREDICTED: similar to transcription factor IIIB 150 [Taeniopygia guttata]
isogroup01805	SOSS complex subunit C [Gallus gallus]
isogroup01811	PREDICTED: survival of motor neuron [Taeniopygia guttata]
isogroup01868	PREDICTED: ankyrin repeat domain protein 15 [Gallus gallus]
isogroup01902	PREDICTED: NADH dehydrogenase (ubiquinone) Fe-S protein 4 [Taeniopygia guttata]
isogroup01948	PREDICTED: ST8 alpha-N-acetyl-neuraminide alpha-2,8-sialyltransferase 5 [Taeniopygia guttata]
isogroup02104	PREDICTED: follistatin [Taeniopygia guttata]
isogroup02116	PREDICTED: UDP-N-acetylglucosamine-2-epimerase/N-acetylmannosamine kinase [Taeniopygia guttata]
isogroup02203	PREDICTED: transmembrane protein 161B [Taeniopygia guttata]
isogroup02238	PREDICTED: similar to putative peptidylprolyl isomerase C [Taeniopygia guttata]
isogroup02253	PREDICTED: similar to Oxidation resistance 1 [Gallus gallus]
isogroup02286	NA
isogroup02293	PREDICTED: similar to transcription factor IIIB 150 [Taeniopygia guttata]
isogroup02353	PREDICTED: hydroxymethylglutaryl-CoA synthase 1 [Taeniopygia guttata]
isogroup02406	PREDICTED: similar to isochorismatase domain containing 1 [Gallus gallus]
isogroup02529	PREDICTED: similar to FERM and PDZ domain containing 4 [Taeniopygia guttata]
isogroup02549	NA
isogroup02560	unnamed protein product [Homo sapiens]
isogroup02677	mutated in colorectal cancers [Bos taurus]

Isogroup	Annotation from nr Database
isogroup02714	PREDICTED: similar to LOC100170471 protein [Taeniopygia guttata]
isogroup02737	PREDICTED: hypothetical protein [Taeniopygia guttata]
isogroup02901	PREDICTED: similar to Ras-like without CAAX 2 [Taeniopygia guttata]
isogroup02948	PREDICTED: hypothetical protein [Taeniopygia guttata]
isogroup02951	PREDICTED: similar to RAD26L hypothetical protein [Equus caballus]
isogroup02995	PREDICTED: similar to calcium channel, voltage-dependent, alpha 2/delta subunit 1 [Taeniopygia guttata]
isogroup03065	unnamed protein product [Homo sapiens]
isogroup03074	PREDICTED: multiple PDZ domain protein [Taeniopygia guttata]
isogroup03097	PREDICTED: phosphatidylinositol-4-phosphate 5-kinase, type I, beta [Taeniopygia guttata]
isogroup03129	PREDICTED: ring finger protein 20 [Taeniopygia guttata]
isogroup03165	PREDICTED: retinoic acid induced 14 [Taeniopygia guttata]
isogroup03176	PREDICTED: similar to polyadenylate binding protein-interacting protein 1 [Taeniopygia guttata]
isogroup03311	PREDICTED: importin 11 [Taeniopygia guttata]
isogroup03336	PREDICTED: PC4 and SFRS1 interacting protein 1 [Taeniopygia guttata]
isogroup03358	PREDICTED: similar to EEF2K protein [Taeniopygia guttata]
isogroup03360	putative fructosamine-3-kinase-related protein [Taeniopygia guttata]
isogroup03383	PREDICTED: similar to UBQLN1 protein [Gallus gallus]
isogroup03392	PREDICTED: dedicator of cytokinesis 4 [Taeniopygia guttata]
isogroup03406	PREDICTED: similar to ELAV (embryonic lethal, abnormal vision, Drosophila)-like 2 (Hu antigen B) (AKA Mel-N1), partial [Gallus gallus]
isogroup03424	PREDICTED: polo-like kinase 2 (Drosophila) [Taeniopygia guttata]
isogroup03475	NA
isogroup03525	PREDICTED: fukutin [Taeniopygia guttata]
isogroup03540	histone H2A.Z [Gallus gallus]
isogroup03572	PREDICTED: arrestin domain containing 3 [Taeniopygia guttata]
isogroup03596	PREDICTED: adenomatous polyposis coli [Taeniopygia guttata]
isogroup03651	PREDICTED: hypothetical protein [Gallus gallus]
isogroup03694	PREDICTED: similar to MEK kinase 1 [Gallus gallus]
isogroup03722	PREDICTED: very low density lipoprotein receptor [Taeniopygia guttata]
isogroup03812	NA
isogroup03926	PREDICTED: serologically defined colon cancer antigen 10 [Taeniopygia guttata]
isogroup03958	SWI/SNF related, matrix associated, actin dependent regulator of chromatin, subfamily a, member 2 [Gallus gallus]
isogroup04073	PREDICTED: similar to ribosomal protein S6 [Taeniopygia guttata]

Isogroup	Annotation from nr Database
isogroup04126	PREDICTED: hypothetical protein [Gallus gallus]
isogroup04163	PREDICTED: acetyl-coenzyme A acyltransferase 2 [Taeniopygia guttata]
isogroup04182	selenoprotein P precursor [Gallus gallus]
isogroup04199	PREDICTED: putative clathrin light polypeptide variant 1a [Taeniopygia guttata]
isogroup04230	PREDICTED: myosin, light chain kinase [Taeniopygia guttata]
isogroup04250	PREDICTED: putative sorting nexin 24 [Taeniopygia guttata]
isogroup04273	casein kinase 1, gamma 3, isoform CRA_c [Homo sapiens]
isogroup04423	PREDICTED: similar to ubiquitin-like with PHD and ring finger domains 2, partial [Taeniopygia guttata]
isogroup04427	NA
isogroup04431	NA
isogroup04464	NA
isogroup04504	NA
isogroup04531	NA
isogroup04581	NA
isogroup04658	NA
isogroup04713	NA
isogroup04723	PREDICTED: WD repeat domain 70 [Taeniopygia guttata]
isogroup04781	NA
isogroup04828	PREDICTED: cholinergic receptor, nicotinic, beta polypeptide 3 [Taeniopygia guttata]
isogroup04886	NA
isogroup04896	NA
isogroup04966	PREDICTED: serine palmitoyltransferase, long chain base subunit 1 [Taeniopygia guttata]
isogroup04990	PREDICTED: similar to phospholipase A2-activating protein [Taeniopygia guttata]
isogroup05006	PREDICTED: adenylate kinase 5 [Taeniopygia guttata]
isogroup05017	NA
isogroup05031	NA
isogroup05085	PREDICTED: hypothetical protein [Monodelphis domestica]
isogroup05113	NA
isogroup05170	NA
isogroup05353	PREDICTED: RNA U, small nuclear RNA export adaptor (phosphorylation regulated) [Taeniopygia guttata]
isogroup05392	PREDICTED: hypothetical protein [Taeniopygia guttata]
isogroup05427	mitogen-activated protein kinase 1 [Gallus gallus]
isogroup05515	PREDICTED: nucleoporin 155kDa [Taeniopygia guttata]

Isogroup	Annotation from nr Database
isogroup05531	NA
isogroup05609	NA
isogroup05708	PREDICTED: dymeclin [Taeniopygia guttata]
isogroup05713	PREDICTED: similar to nudix (nucleoside diphosphate linked moiety X)-type motif 12 [Taeniopygia guttata]
isogroup05773	NA
isogroup05797	PREDICTED: multiple PDZ domain protein [Taeniopygia guttata]
isogroup05834	PREDICTED: hypothetical protein [Taeniopygia guttata]
isogroup05869	NA
isogroup05913	PREDICTED: microtubule-associated protein 1B isoform 1 [Taeniopygia guttata]
isogroup05914	PREDICTED: microtubule-associated protein 1B isoform 1 [Taeniopygia guttata]
isogroup05915	NA
isogroup05930	PREDICTED: KIAA0020 [Taeniopygia guttata]
isogroup06021	PREDICTED: synuclein, alpha interacting protein (synphilin) [Taeniopygia guttata]
isogroup06094	PREDICTED: myotubularin related protein 6 [Taeniopygia guttata]
isogroup06113	PREDICTED: similar to ADP-ribosylation factor-like 15 [Taeniopygia guttata]
isogroup06169	PREDICTED: tudor domain containing 7 [Taeniopygia guttata]
isogroup06200	NA
isogroup06263	PREDICTED: adenomatous polyposis coli [Taeniopygia guttata]
isogroup06318	PREDICTED: RIO kinase 2 [Taeniopygia guttata]
isogroup06419	PREDICTED: hypothetical protein [Monodelphis domestica]
isogroup06434	NA
isogroup06501	PREDICTED: microtubule-associated protein 1B isoform 2 [Taeniopygia guttata]
isogroup06512	NA
isogroup06549	purpurin precursor [Gallus gallus]
isogroup06580	PREDICTED: erythrocyte membrane protein band 4.1 like 4A [Taeniopygia guttata]
isogroup06637	PREDICTED: Dmx-like 1 [Taeniopygia guttata]
isogroup06885	NA
isogroup06899	PREDICTED: serum response factor binding protein 1 [Taeniopygia guttata]
isogroup06908	NA
isogroup06935	E3 ubiquitin-protein ligase BRE1A [Gallus gallus]
isogroup07161	PREDICTED: similar to RING-finger protein [Taeniopygia guttata]
isogroup07178	NA
isogroup07209	PREDICTED: similar to adaptor protein, phosphotyrosine interaction, PH domain and leucine zipper containing 1 [Taeniopygia guttata]
isogroup07263	PREDICTED: KIAA0368 [Taeniopygia guttata]

Isogroup	Annotation from nr Database
isogroup07269	NA
isogroup07298	PREDICTED: family with sequence similarity 108, member B1 [Taeniopygia guttata]
isogroup07387	NA
isogroup07415	NA
isogroup07433	NA
isogroup07467	PREDICTED: interferon regulatory factor 1 [Taeniopygia guttata]
isogroup07587	PREDICTED: leukemia inhibitory factor receptor [Taeniopygia guttata]
isogroup07642	NA
isogroup07759	NA
isogroup07867	PREDICTED: DEAD (Asp-Glu-Ala-Asp) box polypeptide 58 [Taeniopygia guttata]
isogroup08004	NA
isogroup08103	NA
isogroup08147	PREDICTED: hypothetical protein [Taeniopygia guttata]
isogroup08222	NA
isogroup08231	NA
isogroup08266	NA
isogroup08325	PREDICTED: YTH domain containing 2 [Oryctolagus cuniculus]
isogroup08358	PREDICTED: hypothetical protein [Taeniopygia guttata]
isogroup08418	PREDICTED: similar to TBC1 domain family member 2A [Taeniopygia guttata]
isogroup08448	PREDICTED: IQ motif containing E [Taeniopygia guttata]
isogroup08490	NA
isogroup08621	NA
isogroup08691	NA
isogroup08834	PREDICTED: alpha-L-iduronidase [Taeniopygia guttata]
isogroup08873	NA
isogroup08899	NA
isogroup08971	NA
isogroup09079	NA
isogroup09145	PREDICTED: hypothetical protein [Taeniopygia guttata]
isogroup09184	NA
isogroup09533	PREDICTED: similar to Frataxin, mitochondrial precursor (Friedreich ataxia protein) (Fxn) [Gallus gallus]
isogroup09612	PREDICTED: similar to yeast ribosomal protein S28 homologue [Gallus gallus]
isogroup09920	NA
isogroup10056	NA
isogroup10061	NA

Isogroup	Annotation from nr Database
isogroup10067	PREDICTED: UDP-N-acetylglucosamine-2-epimerase/N-acetylmannosamine kinase [Taeniopygia guttata]
isogroup10103	PREDICTED: hypothetical protein [Taeniopygia guttata]
isogroup10230	NA
isogroup10438	PREDICTED: hypothetical protein [Taeniopygia guttata]
isogroup10596	NA
isogroup10663	NA
isogroup10717	NA
isogroup10743	PREDICTED: patched [Taeniopygia guttata]
isogroup10765	PREDICTED: similar to guanine deaminase [Taeniopygia guttata]
isogroup10795	PREDICTED: hypothetical protein [Taeniopygia guttata]
isogroup10861	PREDICTED: hypothetical protein [Taeniopygia guttata]
isogroup10973	NA
isogroup11007	PREDICTED: pleckstrin and Sec7 domain containing 3 [Taeniopygia guttata]
isogroup11083	PREDICTED: similar to hydroxysteroid dehydrogenase like 2 [Taeniopygia guttata]
isogroup11134	NA
isogroup11150	PREDICTED: similar to KIAA2026 [Pan troglodytes]
isogroup11161	PREDICTED: similar to sushi domain containing 1 [Taeniopygia guttata]
isogroup11177	unnamed protein product [Homo sapiens]
isogroup11231	PREDICTED: similar to KIAA0372 [Gallus gallus]
isogroup11238	PREDICTED: protein inhibitor of activated STAT X [Taeniopygia guttata]
isogroup11418	NA
isogroup11420	PREDICTED: putative clathrin light polypeptide variant 1a isoform 2 [Taeniopygia guttata]
isogroup11422	PREDICTED: similar to MGC84124 protein [Taeniopygia guttata]
isogroup11449	PREDICTED: retinoic acid induced 14 [Taeniopygia guttata]
isogroup11513	PREDICTED: putative Di-Ras2 variant 2 [Taeniopygia guttata]
isogroup11520	PREDICTED: hypothetical protein [Taeniopygia guttata]
isogroup11674	PREDICTED: similar to cAMP responsive element binding protein 3-like 3 [Taeniopygia guttata]
isogroup12016	PREDICTED: dedicator of cytokinesis 4 [Taeniopygia guttata]
isogroup12023	NA
isogroup12033	PREDICTED: similar to phosphodiesterase 8B [Taeniopygia guttata]
isogroup12045	NA
isogroup12077	thioredoxin 1 [Melopsittacus undulatus]
isogroup12080	NA
isogroup12194	PREDICTED: KIAA0258 [Taeniopygia guttata]

Isogroup	Annotation from nr Database
isogroup12217	NA
isogroup12247	aryl hydrocarbon receptor nuclear translocator 2 [Phalacrocorax carbo]
isogroup12248	PREDICTED: 3-hydroxy-3-methylglutaryl-Coenzyme A reductase [Taeniopygia guttata]
isogroup12256	PREDICTED: inhibitor of kappa light polypeptide gene enhancer in B-cells, kinase complex-associated protein [Taeniopygia guttata]
isogroup12315	NA
isogroup12327	PREDICTED: similar to rapamycin-insensitive companion of mTOR [Taeniopygia guttata]
isogroup12492	WD40 repeat-containing protein SMU1 [Gallus gallus]
isogroup12583	PREDICTED: putative dynactin light chain 3 [Taeniopygia guttata]
isogroup12663	PREDICTED: similar to calmin [Taeniopygia guttata]
isogroup12666	PREDICTED: similar to chorea-acanthocytosis [Taeniopygia guttata]
isogroup12744	PREDICTED: similar to serine/threonine-protein kinase NIM1 [Taeniopygia guttata]
isogroup12760	PREDICTED: WD repeat domain 36 [Taeniopygia guttata]
isogroup12781	NA
isogroup12809	PREDICTED: similar to UPF0418 protein FAM164A [Taeniopygia guttata]
isogroup12855	PREDICTED: similar to NDUFA12-like [Gallus gallus]
isogroup12913	PREDICTED: similar to peptidylprolyl isomerase domain and WD repeat containing 1 [Taeniopygia guttata]
isogroup12924	NA
isogroup12934	PREDICTED: fem-1 homolog c (C. elegans) [Taeniopygia guttata]
isogroup12944	NA
isogroup13011	PREDICTED: RNA terminal phosphate cyclase-like 1 [Taeniopygia guttata]
isogroup13022	NA
isogroup13078	NA
isogroup13101	PREDICTED: similar to phosphatidylinositol glycan, class G [Taeniopygia guttata]
isogroup13110	NA
isogroup13208	NA
isogroup13220	NA
isogroup13242	NA
isogroup13247	NA
isogroup13255	PREDICTED: similar to diacylglycerol kinase, theta [Taeniopygia guttata]
isogroup13260	NA
isogroup13285	PREDICTED: adenomatous polyposis coli [Taeniopygia guttata]
isogroup13439	PREDICTED: similar to guanine deaminase [Taeniopygia guttata]
isogroup13498	NA

Isogroup	Annotation from nr Database
isogroup13499	PREDICTED: neurolysin (metallopeptidase M3 family) [Taeniopygia guttata]
isogroup13712	PREDICTED: small glutamine-rich tetratricopeptide repeat (TPR)-containing, beta [Taeniopygia guttata]
isogroup13741	NA
isogroup13761	PREDICTED: putative Adenylate kinase 3 alpha like 1 variant 1 [Taeniopygia guttata]
isogroup13833	NA
isogroup13887	PREDICTED: superkiller viralicidic activity 2-like 2 [Taeniopygia guttata]
isogroup13892	PREDICTED: fucosyltransferase 10 (alpha (1,3) fucosyltransferase) [Taeniopygia guttata]
isogroup13911	NA
isogroup14026	PREDICTED: similar to KIAA1797 [Taeniopygia guttata]
isogroup14060	PREDICTED: gamma-aminobutyric acid (GABA) A receptor, alpha 5 [Taeniopygia guttata]
isogroup14093	PREDICTED: similar to Hsp90 co-chaperone Cdc37-like 1 [Taeniopygia guttata]
isogroup14159	PREDICTED: ubiquitin specific peptidase 13 (isopeptidase T-3) [Taeniopygia guttata]
isogroup14169	PREDICTED: LMBR1 domain containing 2 [Taeniopygia guttata]
isogroup14285	PREDICTED: WD repeat domain 36 [Taeniopygia guttata]
isogroup14287	NA
isogroup14313	PREDICTED: glycine dehydrogenase (decarboxylating) [Taeniopygia guttata]
isogroup14347	PREDICTED: hypothetical protein [Taeniopygia guttata]
isogroup14362	NA
isogroup14383	NA
isogroup14475	PREDICTED: similar to 2210408I21Rik protein [Taeniopygia guttata]
isogroup14499	PREDICTED: hypothetical protein [Taeniopygia guttata]
isogroup14588	PREDICTED: hypothetical protein [Taeniopygia guttata]
isogroup14640	NA
isogroup14645	NA
isogroup14706	PREDICTED: inhibitor of kappa light polypeptide gene enhancer in B-cells, kinase complex-associated protein [Taeniopygia guttata]
isogroup14719	PREDICTED: similar to Katanin p60 ATPase-containing subunit A-like 2 [Taeniopygia guttata]
isogroup14749	PREDICTED: chromosome 9 open reading frame 95 [Taeniopygia guttata]
isogroup14755	PREDICTED: similar to GABA-A receptor alpha-3 subunit [Monodelphis domestica]
isogroup14784	NA
isogroup14801	NA
isogroup14823	PREDICTED: similar to chorea-acanthocytosis [Taeniopygia guttata]

Isogroup	Annotation from nr Database
isogroup14826	NA
isogroup14880	PREDICTED: ankyrin repeat, family A (RFXANK-like), 2 [Taeniopygia guttata]
isogroup14897	PREDICTED: cholinergic receptor, nicotinic, beta polypeptide 3 [Taeniopygia guttata]
isogroup14905	PREDICTED: excision repair cross-complementing rodent repair deficiency, complementation group 8 [Taeniopygia guttata]
isogroup15123	putative autophagy 12-like [Taeniopygia guttata]
isogroup15166	PREDICTED: hypothetical protein [Taeniopygia guttata]
isogroup15225	PREDICTED: haloacid dehalogenase-like hydrolase domain containing 2 [Taeniopygia guttata]
isogroup15289	PREDICTED: KIAA0368 [Taeniopygia guttata]
isogroup15327	NA
isogroup15339	PREDICTED: putative TGF beta-inducible nuclear protein 1 [Taeniopygia guttata]
isogroup15384	methylcrotonoyl-Coenzyme A carboxylase 2 (beta) [Xenopus laevis]
isogroup15454	NA
isogroup15511	NA
isogroup15537	NA
isogroup15564	NA
isogroup15606	NA
isogroup15645	NA
isogroup15684	PREDICTED: similar to solute carrier family 1 (neuronal/epithelial high affinity glutamate transporter, system Xag), member 1 [Taeniopygia guttata]
isogroup15700	PREDICTED: similar to RING-finger protein [Taeniopygia guttata]
isogroup15718	NA
isogroup15800	PREDICTED: hypothetical protein [Gallus gallus]
isogroup15822	PREDICTED: pentatricopeptide repeat domain 2 [Taeniopygia guttata]
isogroup15855	NA
isogroup15857	NA
isogroup15864	cannabinoid receptor 1 (brain) [Taeniopygia guttata]
isogroup15905	PREDICTED: tetratricopeptide repeat domain 33 [Taeniopygia guttata]
isogroup15912	PREDICTED: hypothetical protein [Taeniopygia guttata]
isogroup15918	PREDICTED: corneal wound healing-related protein [Taeniopygia guttata]
isogroup15928	PREDICTED: similar to DnaJ (Hsp40) homolog, subfamily C, member 21 [Taeniopygia guttata]
isogroup15974	PREDICTED: similar to probable glutathione peroxidase 8-B [Taeniopygia guttata]
isogroup15978	PREDICTED: similar to DCP2 decapping enzyme [Taeniopygia guttata]
isogroup15991	PREDICTED: similar to Probable gluconokinase [Taeniopygia guttata]

Isogroup	Annotation from nr Database
isogroup16017	PREDICTED: hypothetical protein [Taeniopygia guttata]
isogroup16094	PREDICTED: hypothetical protein [Gallus gallus]
isogroup16138	NA
isogroup16139	PREDICTED: similar to KIAA1281 protein [Taeniopygia guttata]
isogroup16198	NA
isogroup16213	PREDICTED: corneal wound healing-related protein [Taeniopygia guttata]
isogroup16345	hydroxysteroid (17-beta) dehydrogenase 4 [Gallus gallus]
isogroup16360	PREDICTED: aprataxin [Taeniopygia guttata]
isogroup16414	NA
isogroup16504	NA
isogroup16535	NA
isogroup16568	PREDICTED: neurotrophic tyrosine kinase, receptor, type 2, partial [Taeniopygia guttata]
isogroup16656	PREDICTED: general transcription factor IIH, polypeptide 2, 44kD subunit isoform 8 [Pan troglodytes]
isogroup16657	PREDICTED: farnesyltransferase, CAAX box, alpha [Taeniopygia guttata]
isogroup16723	PREDICTED: LMBR1 domain containing 2 [Taeniopygia guttata]
isogroup16830	NA
isogroup16831	NA
isogroup16879	PREDICTED: similar to Small nuclear RNA activating complex, polypeptide 3 [Gallus gallus]
isogroup16885	NA
isogroup16905	PREDICTED: Janus kinase 2 [Taeniopygia guttata]
isogroup16937	PREDICTED: inositol 1,3,4-triphosphate 5/6 kinase [Taeniopygia guttata]
isogroup16942	PREDICTED: similar to SAC1 suppressor of actin mutations 1-like [Taeniopygia guttata]
isogroup16988	NA
isogroup17022	PREDICTED: protein kinase, AMP-activated, alpha 1 catalytic subunit [Taeniopygia guttata]
isogroup17041	NA
isogroup17083	PREDICTED: similar to AP-3 complex subunit beta-1 [Taeniopygia guttata]
isogroup17092	PREDICTED: similar to protein tyrosine kinase fer isoform 1 [Bos taurus]
isogroup17106	PREDICTED: similar to alpha-L-iduronidase, partial [Taeniopygia guttata]
isogroup17110	NA
isogroup17131	NA
isogroup17170	calcineurin A alpha [Gallus gallus]
isogroup17195	PREDICTED: hypothetical protein [Taeniopygia guttata]

Isogroup	Annotation from nr Database
isogroup17198	PREDICTED: SET binding protein 1 [Taeniopygia guttata]
isogroup17210	PREDICTED: hypothetical protein [Taeniopygia guttata]
isogroup17216	PREDICTED: hypothetical protein [Gallus gallus]
isogroup17243	PREDICTED: transient receptor potential cation channel, subfamily M, member 3 [Taeniopygia guttata]
isogroup17249	PREDICTED: alpha-methylacyl-CoA racemase [Taeniopygia guttata]
isogroup17296	PREDICTED: histidine acid phosphatase domain containing 1 [Taeniopygia guttata]
isogroup17356	PREDICTED: similar to ring finger protein 38 [Monodelphis domestica]
isogroup17371	NA
isogroup17372	talin 1 [Gallus gallus]
isogroup17573	PREDICTED: sema domain, immunoglobulin domain (Ig), transmembrane domain (TM) and short cytoplasmic domain, (semaphorin) 4D [Taeniopygia guttata]
isogroup17575	NA
isogroup17604	sorting nexin 2 [Gallus gallus]
isogroup17755	NA
isogroup17760	NA
isogroup17874	PREDICTED: hypothetical protein [Taeniopygia guttata]
isogroup17888	NA
isogroup17971	NA
isogroup18001	PREDICTED: similar to GC-rich promoter binding protein 1 isoform 2 [Taeniopygia guttata]
isogroup18078	NA
isogroup18135	PREDICTED: hypothetical protein [Taeniopygia guttata]
isogroup18175	NA
isogroup18409	NA
isogroup18440	NA
isogroup18497	putative DnaJ subfamily A member 1 variant 3 [Taeniopygia guttata]
isogroup18526	NA
isogroup18645	NA
isogroup18646	PREDICTED: G protein-coupled receptor 158 [Taeniopygia guttata]
isogroup18693	PREDICTED: adenomatous polyposis coli [Taeniopygia guttata]
isogroup18708	PREDICTED: similar to Hippocampus abundant transcript-like protein 1 [Taeniopygia guttata]
isogroup18739	PREDICTED: hypothetical protein [Taeniopygia guttata]
isogroup18743	PREDICTED: similar to G elongation factor, mitochondrial 2 [Taeniopygia guttata]
isogroup18765	unnamed protein product [Mus musculus]
isogroup18777	PREDICTED: insulin-like growth factor 1 receptor [Taeniopygia guttata]

Isogroup	Annotation from nr Database
isogroup18887	PREDICTED: similar to ring finger protein 38 [Monodelphis domestica]
isogroup18904	NA
isogroup19029	PREDICTED: fer (fps/fes related) tyrosine kinase (phosphoprotein NCP94) [Taeniopygia guttata]
isogroup19083	PREDICTED: latrophilin 3 [Equus caballus]
isogroup19091	PREDICTED: similar to phosphoinositide-3-kinase, regulatory subunit 1 (alpha) [Taeniopygia guttata]
isogroup19131	PREDICTED: similar to mutS homolog 3 [Taeniopygia guttata]
isogroup19305	NA
isogroup19314	NA
isogroup19341	PREDICTED: similar to AGTPBP1 protein [Gallus gallus]
isogroup19416	PREDICTED: neuronal protein 3.1 [Taeniopygia guttata]
isogroup19560	PREDICTED: similar to AGTPBP1 protein [Gallus gallus]
isogroup19565	NA
isogroup19575	NA
isogroup19650	NA
isogroup19738	NA
isogroup19875	PREDICTED: nuclear factor, interleukin 3 regulated [Taeniopygia guttata]
isogroup19912	PREDICTED: similar to solute carrier family 4, sodium bicarbonate transporter-like, member 10 [Ornithorhynchus anatinus]
isogroup19973	PREDICTED: microtubule-associated protein 1B isoform 1 [Taeniopygia guttata]
isogroup20079	PREDICTED: similar to adaptor protein, phosphotyrosine interaction, PH domain and leucine zipper containing 1 [Taeniopygia guttata]
isogroup20211	PREDICTED: similar to arylsulfatase B [Taeniopygia guttata]
isogroup20226	NA
isogroup20237	PREDICTED: centrosomal protein 78kDa [Taeniopygia guttata]
isogroup20295	NA
isogroup20325	NA
isogroup20328	PREDICTED: hypothetical protein [Taeniopygia guttata]
isogroup20368	NA
isogroup20463	PREDICTED: similar to cyclin G associated kinase [Taeniopygia guttata]
isogroup20470	PREDICTED: similar to DnaJ (Hsp40) homolog, subfamily C, member 21 [Taeniopygia guttata]
isogroup20473	PREDICTED: similar to SECIS binding protein 2 [Gallus gallus]
isogroup20504	PREDICTED: ATPase, class II, type 9B [Taeniopygia guttata]
isogroup20549	NA
isogroup20555	PREDICTED: similar to transcription factor IIIB 150 [Taeniopygia guttata]

Isogroup	Annotation from nr Database
isogroup20562	NA
isogroup20660	PREDICTED: similar to megakaryoblastic leukemia 2 protein [Gallus gallus]
isogroup20761	PREDICTED: similar to coagulation factor II (thrombin) receptor [Taeniopygia guttata]
isogroup20772	PREDICTED: hypothetical protein [Taeniopygia guttata]
isogroup20776	PREDICTED: similar to TBC1 domain family member 2A [Taeniopygia guttata]
isogroup20795	PREDICTED: DEAH (Asp-Glu-Ala-His) box polypeptide 29 [Taeniopygia guttata]
isogroup20800	NA
isogroup20817	NA
isogroup20848	PREDICTED: similar to chorea-acanthocytosis [Taeniopygia guttata]
isogroup20898	NA
isogroup20938	NA
isogroup20955	NA
isogroup20993	PREDICTED: similar to oxytocinase splice [Gallus gallus]
isogroup21009	NA
isogroup21015	NA
isogroup21019	PREDICTED: COMM domain containing 10 [Taeniopygia guttata]
isogroup21109	NA
isogroup21177	NA
isogroup21194	PREDICTED: amyloid beta (A4) precursor protein-binding, family A, member 1 [Taeniopygia guttata]
isogroup21279	PREDICTED: jumonji domain containing 2C [Taeniopygia guttata]
isogroup21281	PREDICTED: hypothetical protein [Taeniopygia guttata]
isogroup21321	PREDICTED: similar to monogenic, audiogenic seizure susceptibility 1 homolog (mouse), [Monodelphis domestica]
isogroup21329	PREDICTED: WD repeat domain 41 [Taeniopygia guttata]
isogroup21365	NA
isogroup21373	NA
isogroup21432	NA
isogroup21435	NA
isogroup21454	PREDICTED: interleukin 6 signal transducer (gp130, oncostatin M receptor) [Taeniopygia guttata]
isogroup21598	NA
isogroup21656	PREDICTED: similar to ubiquitin-like with PHD and ring finger domains 2, partial [Taeniopygia guttata]
isogroup21810	PREDICTED: similar to Integrin alpha-2 [Taeniopygia guttata]
isogroup21857	PREDICTED: similar to phosphatidylinositol glycan, class G [Taeniopygia guttata]

Isogroup	Annotation from nr Database
isogroup21872	NA
isogroup21886	PREDICTED: osteoclast stimulating factor 1 [Taeniopygia guttata]
isogroup21923	NA
isogroup21953	NA
isogroup22026	NA
isogroup22037	NA
isogroup22044	PREDICTED: hypothetical protein [Taeniopygia guttata]
isogroup22075	PREDICTED: similar to FCH domain only protein 2 [Taeniopygia guttata]
isogroup22087	hypothetical protein [Taeniopygia guttata]
isogroup22093	PREDICTED: homer 1-like [Danio rerio]
isogroup22217	NA
isogroup22242	PREDICTED: hypothetical protein [Taeniopygia guttata]
isogroup22262	PREDICTED: zinc finger, CCHC domain containing 6 [Taeniopygia guttata]
isogroup22279	NA
isogroup22354	NA
isogroup22374	PREDICTED: hypothetical protein [Taeniopygia guttata]
isogroup22459	NA
isogroup22507	NA
isogroup22560	PREDICTED: solute carrier family 30 (zinc transporter), member 5 [Taeniopygia guttata]
isogroup22576	PREDICTED: hypothetical protein [Gallus gallus]
isogroup22601	PREDICTED: similar to X-ray repair cross complementing protein 4 [Taeniopygia guttata]
isogroup22610	NA
isogroup22614	PREDICTED: similar to karyopherin beta2 [Gallus gallus]
isogroup22617	NA
isogroup22646	NA
isogroup22667	NA
isogroup22714	NA
isogroup22726	PREDICTED: multiple PDZ domain protein [Taeniopygia guttata]

Supplementary Table 5. Genes expressed higher in females than males in the hypothalamus.

Isogroup	Annotation from nr Database
isogroup00008	PREDICTED: similar to UTP18, small subunit processome component [Taeniopygia guttata]
isogroup00017	PREDICTED: similar to Envelope glycoprotein [Taeniopygia guttata]
isogroup00027	PREDICTED: similar to coiled-coil domain containing 5 [Taeniopygia guttata]
isogroup00028	adipocyte differentiation-related protein [Gallus gallus]
isogroup00032	NA
isogroup00052	NA
isogroup00073	NA
isogroup00097	PREDICTED: transforming, acidic coiled-coil containing protein 3 [Taeniopygia guttata]
isogroup00147	NA
isogroup00157	NA
isogroup00230	NA
isogroup00260	PREDICTED: similar to Gag-Pro-Pol-Env protein [Taeniopygia guttata]
isogroup00396	PREDICTED: similar to KIAA1892 protein - DDB1- and CUL4-associated factor 12 [Monodelphis domestica]
isogroup00459	NA
isogroup00499	PREDICTED: hypothetical protein [Taeniopygia guttata]
isogroup00660	PREDICTED: similar to polyprotein [Taeniopygia guttata]
isogroup00829	PREDICTED: collagen, type IV, alpha 3 (Goodpasture antigen) binding protein [Taeniopygia guttata]
isogroup00842	PREDICTED: similar to Gag-Pro-Pol-Env protein [Taeniopygia guttata]
isogroup00849	activated RNA polymerase II transcriptional coactivator p15 [Gallus gallus]
isogroup00907	R envelope protein [Hylobates moloch]
isogroup00912	NA
isogroup00934	PREDICTED: ADP-ribosylation factor binding protein 3 [Taeniopygia guttata]
isogroup00993	PREDICTED: similar to interleukin 22-binding protein [Taeniopygia guttata]
isogroup01222	PREDICTED: similar to pol-like protein ENS-3, partial [Taeniopygia guttata]
isogroup01269	valosin-containing protein [Gallus gallus]
isogroup01578	NA
isogroup01716	PREDICTED: similar to potassium channel modulatory factor 1 [Taeniopygia guttata]
isogroup01746	PREDICTED: zinc finger, SWIM-type containing 6 [Taeniopygia guttata]
isogroup01959	NA
isogroup01965	PREDICTED: similar to dual oxidase 2 [Taeniopygia guttata]
isogroup01999	NA
isogroup02469	putative protein kinase C inhibitor/ASWZ [Taeniopygia guttata]

Isogroup	Annotation from nr Database
isogroup02618	NA
isogroup02820	PREDICTED: solute carrier family 37 (glycerol-3-phosphate transporter), member 3 [Taeniopygia guttata]
isogroup02829	PREDICTED: similar to ARKadia-like 1 isoform 2 [Taeniopygia guttata]
isogroup02863	NA
isogroup03130	NA
isogroup03190	PREDICTED: hypothetical protein [Gallus gallus]
isogroup03210	NA
isogroup03917	NA
isogroup04068	NA
isogroup04099	NA
isogroup04105	NA
isogroup04159	PREDICTED: similar to LOC496251 protein [Taeniopygia guttata]
isogroup04474	NA
isogroup04702	NA
isogroup04710	unnamed protein product [Homo sapiens]
isogroup04792	NA
isogroup04876	NA
isogroup05190	PREDICTED: similar to Gag polyprotein [Taeniopygia guttata]
isogroup05200	NA
isogroup05470	NA
isogroup05650	NA
isogroup05744	PREDICTED: similar to LAG1 longevity assurance homolog 3 [Taeniopygia guttata]
isogroup05793	PREDICTED: similar to polyprotein [Taeniopygia guttata]
isogroup05969	NA
isogroup06064	NA
isogroup06101	PREDICTED: similar to rCG43799 [Taeniopygia guttata]
isogroup06151	NA
isogroup06326	tumor necrosis factor, alpha-induced protein 6 [Gallus gallus]
isogroup06400	YK047_HUMAN RecName: Full=Putative Ig-like domain-containing protein DKFZp686O24166/DKFZp686I21167; Flags: Precursor
isogroup06711	NA
isogroup07013	NA
isogroup08046	NA
isogroup08439	PREDICTED: similar to Spermatogenesis associated 18 [Gallus gallus]
isogroup08540	NA

Isogroup	Annotation from nr Database
isogroup09002	NA
isogroup09204	NA
isogroup09440	PREDICTED: similar to complement C1qA [Ornithorhynchus anatinus]
isogroup09442	NA
isogroup09662	NA
isogroup10476	PREDICTED: similar to Bardet-Biedl syndrome 10 protein homolog [Taeniopygia guttata]
isogroup10572	hypothetical protein LOC422926 [Gallus gallus]
isogroup10667	transcription factor BTF3 isoform 1 [Mus musculus]
isogroup10781	PREDICTED: similar to Gag-Pro-Pol protein [Taeniopygia guttata]
isogroup11168	PREDICTED: similar to gag-pro-pol polyprotein [Taeniopygia guttata]
isogroup11584	PREDICTED: hypothetical protein [Taeniopygia guttata]
isogroup11851	NA
isogroup12363	NA
isogroup12400	PREDICTED: similar to polymerase [Taeniopygia guttata]
isogroup12748	PREDICTED: proteasome alpha 4 subunit-like [Oryctolagus cuniculus]
isogroup13009	NA
isogroup13068	PREDICTED: similar to MEX3C [Ornithorhynchus anatinus]
isogroup13627	NA
isogroup13767	NA
isogroup13902	NA
isogroup14064	PREDICTED: similar to pol-like protein ENS-3, partial [Taeniopygia guttata]
isogroup15188	PREDICTED: similar to panopsin [Gallus gallus]
isogroup15562	NA
isogroup15585	NA
isogroup15653	PREDICTED: similar to pol protein [Ornithorhynchus anatinus]
isogroup15965	NA
isogroup16214	PREDICTED: similar to trans-Golgi protein GMx33 [Gallus gallus]
isogroup16708	NA
isogroup17098	NA
isogroup17167	NA
isogroup17199	POL-like [Gallus gallus]
isogroup17235	NA
isogroup17762	NA
isogroup18358	PREDICTED: similar to zinc finger protein 31 isoform 2 [Macaca mulatta]
isogroup19043	env [Bonasa umbellus]

Isogroup	Annotation from nr Database
isogroup19373	hypothetical protein [Gallus gallus]
isogroup19448	PREDICTED: similar to pol protein [Taeniopygia guttata]
isogroup19487	NA
isogroup19515	NA
isogroup19538	NA
isogroup19731	NA
isogroup19806	NA
isogroup19892	cdc48 [Larimichthys crocea]
isogroup20015	NA
isogroup20209	PREDICTED: hypothetical protein [Taeniopygia guttata]
isogroup20244	NA
isogroup20441	NA
isogroup20679	NA
isogroup20726	PREDICTED: similar to zinc finger protein [Taeniopygia guttata]
isogroup20821	NA
isogroup21188	NA
isogroup21286	NA
isogroup21339	PREDICTED: transmembrane channel-like 3 [Taeniopygia guttata]
isogroup21533	NA
isogroup21763	PREDICTED: similar to Gag-Pro-Pol-Env protein [Taeniopygia guttata]
isogroup21993	NA
isogroup22206	mKIAA1376 protein [Mus musculus]
isogroup22561	PREDICTED: coiled-coil domain containing 77 [Taeniopygia guttata]
isogroup22638	PREDICTED: transient receptor potential cation channel, subfamily M, member 8 [Taeniopygia guttata]
contig90110	NA
contig90185	NA
contig90192	NA

Supplementary Table 6. Gene Ontology terms over-represented among genes significantly differentially expressed between males and females in the hypothalamus.

GO ID	GO Description
GO:0000060	protein import into nucleus, translocation
GO:0000079	regulation of cyclin-dependent protein kinase activity
GO:0001649	osteoblast differentiation
GO:0002376	immune system process
GO:0003002	regionalization
GO:0006665	sphingolipid metabolic process
GO:0006793	phosphorus metabolic process
GO:0006892	post-Golgi vesicle-mediated transport
GO:0006916	anti-apoptosis
GO:0007019	microtubule depolymerization
GO:0007033	vacuole organization
GO:0007043	cell-cell junction assembly
GO:0007166	cell surface receptor signaling pathway
GO:0008154	actin polymerization or depolymerization
GO:0008283	cell proliferation
GO:0009259	ribonucleotide metabolic process
GO:0009636	response to toxin
GO:0009896	positive regulation of catabolic process
GO:0009968	negative regulation of signal transduction
GO:0010243	response to organic nitrogen
GO:0010740	positive regulation of intracellular protein kinase cascade
GO:0010827	regulation of glucose transport
GO:0016125	sterol metabolic process
GO:0019395	fatty acid oxidation
GO:0030073	insulin secretion
GO:0030307	positive regulation of cell growth
GO:0031103	axon regeneration
GO:0031112	positive regulation of microtubule polymerization or depolymerization
GO:0031269	pseudopodium assembly
GO:0031929	TOR signaling cascade
GO:0032611	interleukin-1 beta production
GO:0033205	cell cycle cytokinesis
GO:0033273	response to vitamin
GO:0033554	cellular response to stress

GO ID	GO Description
GO:0042503	tyrosine phosphorylation of Stat3 protein
GO:0043627	response to estrogen stimulus
GO:0045841	negative regulation of mitotic metaphase/anaphase transition
GO:0048871	multicellular organismal homeostasis
GO:0051093	negative regulation of developmental process
GO:0051313	attachment of spindle microtubules to chromosome
GO:0051914	positive regulation of synaptic plasticity by chemical substance
GO:0070507	regulation of microtubule cytoskeleton organization
GO:0004713	protein tyrosine kinase activity
GO:0004896	cytokine receptor activity
GO:0005057	receptor signaling protein activity
GO:0005126	cytokine receptor binding
GO:0008289	lipid binding
GO:0015631	tubulin binding
GO:0016772	transferase activity, transferring phosphorus-containing groups
GO:0016854	racemase and epimerase activity
GO:0017046	peptide hormone binding
GO:0019207	kinase regulator activity
GO:0019900	kinase binding
GO:0019902	phosphatase binding
GO:0050839	cell adhesion molecule binding
GO:0000775	chromosome, centromeric region
GO:0030424	axon
GO:0030427	site of polarized growth
GO:0043025	neuronal cell body
GO:0043296	apical junction complex

Supplementary Table 7. Genes expressed higher in males than females in both medial amygdala and hypothalamus.

Isogroup	Annotation from nr Database
isogroup00224	PREDICTED: chromosome 9 open reading frame 3 [Taeniopygia guttata]
isogroup00365	PREDICTED: similar to DKFZP459P083 protein isoform 1 [Taeniopygia guttata]
isogroup00451	PREDICTED: similar to GRAM domain containing 3 [Taeniopygia guttata]
isogroup00458	SWI/SNF related, matrix associated, actin dependent regulator of chromatin, subfamily a, member 2 [Gallus gallus]
isogroup00524	PREDICTED: hypothetical protein [Taeniopygia guttata]
isogroup00533	DNA-directed DNA polymerase kappa [Gallus gallus]
isogroup00551	RPTOR independent companion of MTOR, complex 2 [Bos taurus]
isogroup00570	single-stranded DNA binding protein 2 [Homo sapiens]
isogroup00694	PREDICTED: prolactin receptor [Taeniopygia guttata]
isogroup00744	PREDICTED: xeroderma pigmentosum, complementation group A [Taeniopygia guttata]
isogroup00779	PREDICTED: SMC5 protein [Taeniopygia guttata]
isogroup00783	PREDICTED: death-associated protein kinase 1 [Taeniopygia guttata]
isogroup00794	PREDICTED: similar to Lipid phosphate phosphohydrolase 1 [Taeniopygia guttata]
isogroup00984	PREDICTED: similar to KIAA0372 [Gallus gallus]
isogroup00991	PREDICTED: jumonji domain containing 2C [Taeniopygia guttata]
isogroup00999	PREDICTED: COBW domain containing protein1 [Taeniopygia guttata]
isogroup01050	PREDICTED: zinc finger, CCHC domain containing 6 [Taeniopygia guttata]
isogroup01106	PREDICTED: similar to SHC (Src homology 2 domain containing) transforming protein 3 [Taeniopygia guttata]
isogroup01181	PREDICTED: similar to phosphoinositide-3-kinase, class 3 [Taeniopygia guttata]
isogroup01284	PREDICTED: RAS p21 protein activator (GTPase activating protein) 1 [Taeniopygia guttata]
isogroup01427	PREDICTED: microtubule-associated protein 1B isoform 1 [Taeniopygia guttata]
isogroup01466	PREDICTED: similar to protein geranylgeranyltransferase type I, beta subunit isoform 1 [Equus caballus]
isogroup01658	Rpl17 protein [Mus musculus]
isogroup01805	SOSS complex subunit C [Gallus gallus]
isogroup01811	PREDICTED: survival of motor neuron [Taeniopygia guttata]
isogroup01868	PREDICTED: ankyrin repeat domain protein 15 [Gallus gallus]
isogroup01902	PREDICTED: NADH dehydrogenase (ubiquinone) Fe-S protein 4 [Taeniopygia guttata]
isogroup01948	PREDICTED: ST8 alpha-N-acetyl-neuraminide alpha-2,8-sialyltransferase 5 [Taeniopygia guttata]
isogroup02203	PREDICTED: transmembrane protein 161B [Taeniopygia guttata]
isogroup02238	PREDICTED: similar to putative peptidylprolyl isomerase C [Taeniopygia guttata]

Isogroup	Annotation from nr Database
isogroup02286	NA
isogroup02549	NA
isogroup02560	unnamed protein product [Homo sapiens]
isogroup02714	PREDICTED: similar to LOC100170471 protein [Taeniopygia guttata]
isogroup02948	PREDICTED: hypothetical protein [Taeniopygia guttata]
isogroup02951	PREDICTED: similar to RAD26L hypothetical protein [Equus caballus]
isogroup03074	PREDICTED: multiple PDZ domain protein [Taeniopygia guttata]
isogroup03097	PREDICTED: phosphatidylinositol-4-phosphate 5-kinase, type I, beta [Taeniopygia guttata]
isogroup03129	PREDICTED: ring finger protein 20 [Taeniopygia guttata]
isogroup03176	PREDICTED: similar to polyadenylate binding protein-interacting protein 1 [Taeniopygia guttata]
isogroup03336	PREDICTED: PC4 and SFRS1 interacting protein 1 [Taeniopygia guttata]
isogroup03383	PREDICTED: similar to UBQLN1 protein [Gallus gallus]
isogroup03406	PREDICTED: similar to ELAV (embryonic lethal, abnormal vision, Drosophila)-like 2 (Hu antigen B) (AKA Mel-N1), partial [Gallus gallus]
isogroup03572	PREDICTED: arrestin domain containing 3 [Taeniopygia guttata]
isogroup03596	PREDICTED: adenomatous polyposis coli [Taeniopygia guttata]
isogroup03651	PREDICTED: hypothetical protein [Gallus gallus]
isogroup03694	PREDICTED: similar to MEK kinase 1 [Gallus gallus]
isogroup03812	NA
isogroup03958	SWI/SNF related, matrix associated, actin dependent regulator of chromatin, subfamily a, member 2 [Gallus gallus]
isogroup04163	PREDICTED: acetyl-coenzyme A acyltransferase 2 [Taeniopygia guttata]
isogroup04199	PREDICTED: putative clathrin light polypeptide variant 1a [Taeniopygia guttata]
isogroup04250	PREDICTED: putative sorting nexin 24 [Taeniopygia guttata]
isogroup04504	NA
isogroup04531	NA
isogroup04581	NA
isogroup04723	PREDICTED: WD repeat domain 70 [Taeniopygia guttata]
isogroup04781	NA
isogroup04828	PREDICTED: cholinergic receptor, nicotinic, beta polypeptide 3 [Taeniopygia guttata]
isogroup04886	NA
isogroup04966	PREDICTED: serine palmitoyltransferase, long chain base subunit 1 [Taeniopygia guttata]
isogroup04990	PREDICTED: similar to phospholipase A2-activating protein [Taeniopygia guttata]
isogroup05085	PREDICTED: hypothetical protein [Monodelphis domestica]

Isogroup	Annotation from nr Database
isogroup05113	NA
isogroup05531	NA
isogroup05713	PREDICTED: similar to nudix (nucleoside diphosphate linked moiety X)-type motif 12 [Taeniopygia guttata]
isogroup05797	PREDICTED: multiple PDZ domain protein [Taeniopygia guttata]
isogroup05913	PREDICTED: microtubule-associated protein 1B isoform 1 [Taeniopygia guttata]
isogroup05914	PREDICTED: microtubule-associated protein 1B isoform 1 [Taeniopygia guttata]
isogroup05930	PREDICTED: KIAA0020 [Taeniopygia guttata]
isogroup06021	PREDICTED: synuclein, alpha interacting protein (synphilin) [Taeniopygia guttata]
isogroup06263	PREDICTED: adenomatous polyposis coli [Taeniopygia guttata]
isogroup06318	PREDICTED: RIO kinase 2 [Taeniopygia guttata]
isogroup06501	PREDICTED: microtubule-associated protein 1B isoform 2 [Taeniopygia guttata]
isogroup06512	NA
isogroup06899	PREDICTED: serum response factor binding protein 1 [Taeniopygia guttata]
isogroup06935	E3 ubiquitin-protein ligase BRE1A [Gallus gallus]
isogroup07161	PREDICTED: similar to RING-finger protein [Taeniopygia guttata]
isogroup07178	NA
isogroup07263	PREDICTED: KIAA0368 [Taeniopygia guttata]
isogroup07269	NA
isogroup07298	PREDICTED: family with sequence similarity 108, member B1 [Taeniopygia guttata]
isogroup07387	NA
isogroup07587	PREDICTED: leukemia inhibitory factor receptor [Taeniopygia guttata]
isogroup07867	PREDICTED: DEAD (Asp-Glu-Ala-Asp) box polypeptide 58 [Taeniopygia guttata]
isogroup08103	NA
isogroup08222	NA
isogroup08266	NA
isogroup08325	PREDICTED: YTH domain containing 2 [Oryctolagus cuniculus]
isogroup08418	PREDICTED: similar to TBC1 domain family member 2A [Taeniopygia guttata]
isogroup08448	PREDICTED: IQ motif containing E [Taeniopygia guttata]
isogroup08490	NA
isogroup08621	NA
isogroup08834	PREDICTED: alpha-L-iduronidase [Taeniopygia guttata]
isogroup08873	NA
isogroup08899	NA
isogroup08971	NA
isogroup09612	PREDICTED: similar to yeast ribosomal protein S28 homologue [Gallus gallus]

Isogroup	Annotation from nr Database
isogroup09920	NA
isogroup10103	PREDICTED: hypothetical protein [Taeniopygia guttata]
isogroup10438	PREDICTED: hypothetical protein [Taeniopygia guttata]
isogroup10596	NA
isogroup10717	NA
isogroup10743	PREDICTED: patched [Taeniopygia guttata]
isogroup11007	PREDICTED: pleckstrin and Sec7 domain containing 3 [Taeniopygia guttata]
isogroup11083	PREDICTED: similar to hydroxysteroid dehydrogenase like 2 [Taeniopygia guttata]
isogroup11134	NA
isogroup11150	PREDICTED: similar to KIAA2026 [Pan troglodytes]
isogroup11177	unnamed protein product [Homo sapiens]
isogroup11231	PREDICTED: similar to KIAA0372 [Gallus gallus]
isogroup11238	PREDICTED: protein inhibitor of activated STAT X [Taeniopygia guttata]
isogroup11420	PREDICTED: putative clathrin light polypeptide variant 1a isoform 2 [Taeniopygia guttata]
isogroup11520	PREDICTED: hypothetical protein [Taeniopygia guttata]
isogroup11674	PREDICTED: similar to cAMP responsive element binding protein 3-like 3 [Taeniopygia guttata]
isogroup12023	NA
isogroup12033	PREDICTED: similar to phosphodiesterase 8B [Taeniopygia guttata]
isogroup12077	thioredoxin 1 [Melopsittacus undulatus]
isogroup12194	PREDICTED: KIAA0258 [Taeniopygia guttata]
isogroup12217	NA
isogroup12256	PREDICTED: inhibitor of kappa light polypeptide gene enhancer in B-cells, kinase complex-associated protein [Taeniopygia guttata]
isogroup12315	NA
isogroup12327	PREDICTED: similar to rapamycin-insensitive companion of mTOR [Taeniopygia guttata]
isogroup12583	PREDICTED: putative dynactin light chain 3 [Taeniopygia guttata]
isogroup12666	PREDICTED: similar to chorea-acanthocytosis [Taeniopygia guttata]
isogroup12760	PREDICTED: WD repeat domain 36 [Taeniopygia guttata]
isogroup12855	PREDICTED: similar to NDUFA12-like [Gallus gallus]
isogroup12913	PREDICTED: similar to peptidylprolyl isomerase domain and WD repeat containing 1 [Taeniopygia guttata]
isogroup12934	PREDICTED: fem-1 homolog c (C. elegans) [Taeniopygia guttata]
isogroup13022	NA
isogroup13101	PREDICTED: similar to phosphatidylinositol glycan, class G [Taeniopygia guttata]

Isogroup	Annotation from nr Database
isogroup13110	NA
isogroup13220	NA
isogroup13247	NA
isogroup13255	PREDICTED: similar to diacylglycerol kinase, theta [Taeniopygia guttata]
isogroup13285	PREDICTED: adenomatous polyposis coli [Taeniopygia guttata]
isogroup13499	PREDICTED: neurolysin (metallopeptidase M3 family) [Taeniopygia guttata]
isogroup13712	PREDICTED: small glutamine-rich tetratricopeptide repeat (TPR)-containing, beta [Taeniopygia guttata]
isogroup13741	NA
isogroup13761	PREDICTED: putative Adenylate kinase 3 alpha like 1 variant 1 [Taeniopygia guttata]
isogroup13887	PREDICTED: superkiller viralicidic activity 2-like 2 [Taeniopygia guttata]
isogroup13892	PREDICTED: fucosyltransferase 10 (alpha (1,3) fucosyltransferase) [Taeniopygia guttata]
isogroup13911	NA
isogroup14285	PREDICTED: WD repeat domain 36 [Taeniopygia guttata]
isogroup14475	PREDICTED: similar to 2210408I21Rik protein [Taeniopygia guttata]
isogroup14499	PREDICTED: hypothetical protein [Taeniopygia guttata]
isogroup14706	PREDICTED: inhibitor of kappa light polypeptide gene enhancer in B-cells, kinase complex-associated protein [Taeniopygia guttata]
isogroup14719	PREDICTED: similar to Katanin p60 ATPase-containing subunit A-like 2 [Taeniopygia guttata]
isogroup14749	PREDICTED: chromosome 9 open reading frame 95 [Taeniopygia guttata]
isogroup14784	NA
isogroup14801	NA
isogroup14823	PREDICTED: similar to chorea-acanthocytosis [Taeniopygia guttata]
isogroup14880	PREDICTED: ankyrin repeat, family A (RFXANK-like), 2 [Taeniopygia guttata]
isogroup14905	PREDICTED: excision repair cross-complementing rodent repair deficiency, complementation group 8 [Taeniopygia guttata]
isogroup15123	putative autophagy 12-like [Taeniopygia guttata]
isogroup15166	PREDICTED: hypothetical protein [Taeniopygia guttata]
isogroup15225	PREDICTED: haloacid dehalogenase-like hydrolase domain containing 2 [Taeniopygia guttata]
isogroup15289	PREDICTED: KIAA0368 [Taeniopygia guttata]
isogroup15384	methylcrotonoyl-Coenzyme A carboxylase 2 (beta) [Xenopus laevis]
isogroup15511	NA
isogroup15537	NA
isogroup15645	NA
isogroup15684	PREDICTED: similar to solute carrier family 1 (neuronal/epithelial high affinity

Isogroup	Annotation from nr Database
	glutamate transporter, system Xag), member 1 [Taeniopygia guttata]
isogroup15700	PREDICTED: similar to RING-finger protein [Taeniopygia guttata]
isogroup15718	NA
isogroup15800	PREDICTED: hypothetical protein [Gallus gallus]
isogroup15822	PREDICTED: pentatricopeptide repeat domain 2 [Taeniopygia guttata]
isogroup15855	NA
isogroup15857	NA
isogroup15905	PREDICTED: tetratricopeptide repeat domain 33 [Taeniopygia guttata]
isogroup15918	PREDICTED: corneal wound healing-related protein [Taeniopygia guttata]
isogroup15928	PREDICTED: similar to DnaJ (Hsp40) homolog, subfamily C, member 21 [Taeniopygia guttata]
isogroup16017	PREDICTED: hypothetical protein [Taeniopygia guttata]
isogroup16139	PREDICTED: similar to KIAA1281 protein [Taeniopygia guttata]
isogroup16198	NA
isogroup16213	PREDICTED: corneal wound healing-related protein [Taeniopygia guttata]
isogroup16345	hydroxysteroid (17-beta) dehydrogenase 4 [Gallus gallus]
isogroup16360	PREDICTED: aprataxin [Taeniopygia guttata]
isogroup16535	NA
isogroup16568	PREDICTED: neurotrophic tyrosine kinase, receptor, type 2, partial [Taeniopygia guttata]
isogroup16656	PREDICTED: general transcription factor IIH, polypeptide 2, 44kD subunit isoform 8 [Pan troglodytes]
isogroup16723	PREDICTED: LMBR1 domain containing 2 [Taeniopygia guttata]
isogroup16831	NA
isogroup16879	PREDICTED: similar to Small nuclear RNA activating complex, polypeptide 3 [Gallus gallus]
isogroup16885	NA
isogroup16937	PREDICTED: inositol 1,3,4-triphosphate 5/6 kinase [Taeniopygia guttata]
isogroup17083	PREDICTED: similar to AP-3 complex subunit beta-1 [Taeniopygia guttata]
isogroup17092	PREDICTED: similar to protein tyrosine kinase fer isoform 1 [Bos taurus]
isogroup17106	PREDICTED: similar to alpha-L-iduronidase, partial [Taeniopygia guttata]
isogroup17110	NA
isogroup17131	NA
isogroup17195	PREDICTED: hypothetical protein [Taeniopygia guttata]
isogroup17198	PREDICTED: SET binding protein 1 [Taeniopygia guttata]
isogroup17210	PREDICTED: hypothetical protein [Taeniopygia guttata]
isogroup17243	PREDICTED: transient receptor potential cation channel, subfamily M, member 3

Isogroup	Annotation from nr Database
	[Taeniopygia guttata]
isogroup17296	PREDICTED: histidine acid phosphatase domain containing 1 [Taeniopygia guttata]
isogroup17356	PREDICTED: similar to ring finger protein 38 [Monodelphis domestica]
isogroup17372	talin 1 [Gallus gallus]
isogroup17573	PREDICTED: sema domain, immunoglobulin domain (Ig), transmembrane domain (TM) and short cytoplasmic domain, (semaphorin) 4D [Taeniopygia guttata]
isogroup17604	sorting nexin 2 [Gallus gallus]
isogroup17874	PREDICTED: hypothetical protein [Taeniopygia guttata]
isogroup17888	NA
isogroup17971	NA
isogroup18135	PREDICTED: hypothetical protein [Taeniopygia guttata]
isogroup18497	putative DnaJ subfamily A member 1 variant 3 [Taeniopygia guttata]
isogroup18645	NA
isogroup18708	PREDICTED: similar to Hippocampus abundant transcript-like protein 1 [Taeniopygia guttata]
isogroup18739	PREDICTED: hypothetical protein [Taeniopygia guttata]
isogroup18743	PREDICTED: similar to G elongation factor, mitochondrial 2 [Taeniopygia guttata]
isogroup18777	PREDICTED: insulin-like growth factor 1 receptor [Taeniopygia guttata]
isogroup18887	PREDICTED: similar to ring finger protein 38 [Monodelphis domestica]
isogroup18904	NA
isogroup19029	PREDICTED: fer (fps/fes related) tyrosine kinase (phosphoprotein NCP94) [Taeniopygia guttata]
isogroup19416	PREDICTED: neuronal protein 3.1 [Taeniopygia guttata]
isogroup19565	NA
isogroup19575	NA
isogroup19650	NA
isogroup20211	PREDICTED: similar to arylsulfatase B [Taeniopygia guttata]
isogroup20237	PREDICTED: centrosomal protein 78kDa [Taeniopygia guttata]
isogroup20295	NA
isogroup20328	PREDICTED: hypothetical protein [Taeniopygia guttata]
isogroup20463	PREDICTED: similar to cyclin G associated kinase [Taeniopygia guttata]
isogroup20470	PREDICTED: similar to DnaJ (Hsp40) homolog, subfamily C, member 21 [Taeniopygia guttata]
isogroup20549	NA
isogroup20555	PREDICTED: similar to transcription factor IIIB 150 [Taeniopygia guttata]
isogroup20562	NA
isogroup20772	PREDICTED: hypothetical protein [Taeniopygia guttata]

Isogroup	Annotation from nr Database
isogroup20776	PREDICTED: similar to TBC1 domain family member 2A [Taeniopygia guttata]
isogroup20800	NA
isogroup20938	NA
isogroup20993	PREDICTED: similar to oxytocinase splice [Gallus gallus]
isogroup21015	NA
isogroup21194	PREDICTED: amyloid beta (A4) precursor protein-binding, family A, member 1 [Taeniopygia guttata]
isogroup21279	PREDICTED: jumonji domain containing 2C [Taeniopygia guttata]
isogroup21281	PREDICTED: hypothetical protein [Taeniopygia guttata]
isogroup21321	PREDICTED: similar to monogenic, audiogenic seizure susceptibility 1 homolog (mouse), [Monodelphis domestica]
isogroup21329	PREDICTED: WD repeat domain 41 [Taeniopygia guttata]
isogroup21454	PREDICTED: interleukin 6 signal transducer (gp130, oncostatin M receptor) [Taeniopygia guttata]
isogroup21656	PREDICTED: similar to ubiquitin-like with PHD and ring finger domains 2, partial [Taeniopygia guttata]
isogroup21857	PREDICTED: similar to phosphatidylinositol glycan, class G [Taeniopygia guttata]
isogroup21886	PREDICTED: osteoclast stimulating factor 1 [Taeniopygia guttata]
isogroup21953	NA
isogroup22026	NA
isogroup22037	NA
isogroup22044	PREDICTED: hypothetical protein [Taeniopygia guttata]
isogroup22075	PREDICTED: similar to FCH domain only protein 2 [Taeniopygia guttata]
isogroup22087	hypothetical protein [Taeniopygia guttata]
isogroup22217	NA
isogroup22262	PREDICTED: zinc finger, CCHC domain containing 6 [Taeniopygia guttata]
isogroup22507	NA
isogroup22576	PREDICTED: hypothetical protein [Gallus gallus]
isogroup22601	PREDICTED: similar to X-ray repair cross complementing protein 4 [Taeniopygia guttata]
isogroup22667	NA
isogroup22714	NA
isogroup22726	PREDICTED: multiple PDZ domain protein [Taeniopygia guttata]

Supplementary Table 8. Genes expressed higher in females than males in both medial amygdala and hypothalamus.

Isogroup	Annotation from nr Database
isogroup00008	PREDICTED: similar to UTP18, small subunit processome component [Taeniopygia guttata]
isogroup00027	PREDICTED: similar to coiled-coil domain containing 5 [Taeniopygia guttata]
isogroup00028	adipocyte differentiation-related protein [Gallus gallus]
isogroup00032	NA
isogroup00052	NA
isogroup00073	NA
isogroup00097	PREDICTED: transforming, acidic coiled-coil containing protein 3 [Taeniopygia guttata]
isogroup00147	NA
isogroup00230	NA
isogroup00260	PREDICTED: similar to Gag-Pro-Pol-Env protein [Taeniopygia guttata]
isogroup00396	PREDICTED: similar to KIAA1892 protein - DDB1- and CUL4-associated factor 12 [Monodelphis domestica]
isogroup00459	NA
isogroup00499	PREDICTED: hypothetical protein [Taeniopygia guttata]
isogroup00660	PREDICTED: similar to polyprotein [Taeniopygia guttata]
isogroup00829	PREDICTED: collagen, type IV, alpha 3 (Goodpasture antigen) binding protein [Taeniopygia guttata]
isogroup00842	PREDICTED: similar to Gag-Pro-Pol-Env protein [Taeniopygia guttata]
isogroup00849	activated RNA polymerase II transcriptional coactivator p15 [Gallus gallus]
isogroup00993	PREDICTED: similar to interleukin 22-binding protein [Taeniopygia guttata]
isogroup01222	PREDICTED: similar to pol-like protein ENS-3, partial [Taeniopygia guttata]
isogroup01269	valosin-containing protein [Gallus gallus]
isogroup01578	NA
isogroup01716	PREDICTED: similar to potassium channel modulatory factor 1 [Taeniopygia guttata]
isogroup01999	NA
isogroup02469	putative protein kinase C inhibitor/ASWZ [Taeniopygia guttata]
isogroup02829	PREDICTED: similar to ARKadia-like 1 isoform 2 [Taeniopygia guttata]
isogroup02863	NA
isogroup03130	NA
isogroup03190	PREDICTED: hypothetical protein [Gallus gallus]
isogroup03917	NA
isogroup04099	NA
isogroup04474	NA
isogroup04702	NA

Isogroup	Annotation from nr Database
isogroup04710	unnamed protein product [Homo sapiens]
isogroup04876	NA
isogroup05190	PREDICTED: similar to Gag polyprotein [Taeniopygia guttata]
isogroup05470	NA
isogroup05793	PREDICTED: similar to polyprotein [Taeniopygia guttata]
isogroup06101	PREDICTED: similar to rCG43799 [Taeniopygia guttata]
isogroup06151	NA
isogroup06326	tumor necrosis factor, alpha-induced protein 6 [Gallus gallus]
isogroup06400	YK047_HUMAN RecName: Full=Putative Ig-like domain-containing protein DKFZp686O24166/DKFZp686I21167; Flags: Precursor
isogroup08046	NA
isogroup08540	NA
isogroup09204	NA
isogroup10572	hypothetical protein LOC422926 [Gallus gallus]
isogroup10667	transcription factor BTF3 isoform 1 [Mus musculus]
isogroup10781	PREDICTED: similar to Gag-Pro-Pol protein [Taeniopygia guttata]
isogroup11168	PREDICTED: similar to gag-pro-pol polyprotein [Taeniopygia guttata]
isogroup12400	PREDICTED: similar to polymerase [Taeniopygia guttata]
isogroup13068	PREDICTED: similar to MEX3C [Ornithorhynchus anatinus]
isogroup13902	NA
isogroup14064	PREDICTED: similar to pol-like protein ENS-3, partial [Taeniopygia guttata]
isogroup15585	NA
isogroup15653	PREDICTED: similar to pol protein [Ornithorhynchus anatinus]
isogroup16214	PREDICTED: similar to trans-Golgi protein GMx33 [Gallus gallus]
isogroup17167	NA
isogroup17199	POL-like [Gallus gallus]
isogroup17235	NA
isogroup17762	NA
isogroup19043	env [Bonasa umbellus]
isogroup19373	hypothetical protein [Gallus gallus]
isogroup19448	PREDICTED: similar to pol protein [Taeniopygia guttata]
isogroup19487	NA
isogroup19515	NA
isogroup19731	NA
isogroup19892	cdc48 [Larimichthys crocea]
isogroup20441	NA

Isogroup	Annotation from nr Database
isogroup21286	NA
isogroup21763	PREDICTED: similar to Gag-Pro-Pol-Env protein [Taeniopygia guttata]
isogroup22206	mKIAA1376 protein [Mus musculus]
contig90185	NA

Supplementary Table 9. Genes expressed higher in T-treated than control females in the medial amygdala.

Isogroup	Annotation from nr Database
isogroup00208	PREDICTED: similar to KIAA1486 protein [Taeniopygia guttata]
isogroup00574	PREDICTED: similar to semaphorin 5A [Taeniopygia guttata]
isogroup00786	PREDICTED: hypothetical protein [Taeniopygia guttata]
isogroup00865	PREDICTED: glutamate receptor, metabotropic 8 [Taeniopygia guttata]
isogroup01057	PREDICTED: similar to 1-phosphatidylinositol-4,5-bisphosphate phosphodiesterase beta-4 isoform 1 [Taeniopygia guttata]
isogroup01108	PREDICTED: similar to KIAA1622 protein [Taeniopygia guttata]
isogroup01547	NA
isogroup01555	NA
isogroup01574	PREDICTED: splicing factor, arginine/serine-rich 5 [Taeniopygia guttata]
isogroup01755	PREDICTED: hypothetical protein [Gallus gallus]
isogroup01861	NA
isogroup01927	PREDICTED: similar to TFIIS central domain-containing protein 1 [Taeniopygia guttata]
isogroup01948	PREDICTED: ST8 alpha-N-acetyl-neuraminide alpha-2,8-sialyltransferase 5 [Taeniopygia guttata]
isogroup01988	PREDICTED: oxysterol binding protein-like 6 [Taeniopygia guttata]
isogroup02108	PREDICTED: NEDD4 binding protein 2 [Taeniopygia guttata]
isogroup02257	regulator of G-protein signaling 7, isoform CRA_b [Rattus norvegicus]
isogroup02602	PREDICTED: slit homolog 3 (Drosophila) [Taeniopygia guttata]
isogroup02712	NA
isogroup02725	neurexin 3 beta precursor [Gallus gallus]
isogroup02775	PREDICTED: similar to synaptic vesicle glycoprotein 2b [Taeniopygia guttata]
isogroup02899	PREDICTED: similar to IIDS6411 [Gallus gallus]
isogroup02948	PREDICTED: hypothetical protein [Taeniopygia guttata]
isogroup03155	PREDICTED: similar to rCG33261 [Taeniopygia guttata]
isogroup03256	PREDICTED: similar to KIF27A [Monodelphis domestica]
isogroup03412	NA
isogroup03607	PREDICTED: zinc finger protein 533 [Taeniopygia guttata]
isogroup03626	PREDICTED: GRIP and coiled-coil domain containing 2 [Taeniopygia guttata]
isogroup03976	PREDICTED: similar to inositol 1,4,5-trisphosphate 3-kinase B [Taeniopygia guttata]
isogroup04019	PREDICTED: dystonin [Taeniopygia guttata]
isogroup04106	NA
isogroup04503	NA
isogroup04591	NA

Isogroup	Annotation from nr Database
isogroup04607	PREDICTED: thyroid hormone receptor interactor 11 [Taeniopygia guttata]
isogroup04886	NA
isogroup04913	NA
isogroup05028	PREDICTED: reelin [Taeniopygia guttata]
isogroup05209	PREDICTED: neural precursor cell expressed, developmentally down-regulated 1 [Taeniopygia guttata]
isogroup05219	PREDICTED: similar to eukaryotic translation initiation factor 4 gamma, 3 [Gallus gallus]
isogroup05426	PREDICTED: similar to B-cell CLL/lymphoma 9 [Gallus gallus]
isogroup05470	NA
isogroup05882	PREDICTED: nebulette [Macaca mulatta]
isogroup05946	NA
isogroup06104	NA
isogroup06163	PREDICTED: similar to KIAA0753 protein [Taeniopygia guttata]
isogroup06266	PREDICTED: lectin, galactoside-binding, soluble, 8 (galectin 8) [Taeniopygia guttata]
isogroup06512	NA
isogroup06956	PREDICTED: similar to Nesprin-1 (Nuclear envelope spectrin repeat protein 1) (Synaptic nuclear envelope protein 1) (Syne-1) (Myocyte nuclear envelope protein 1) (Myne-1) (Enaptin) [Gallus gallus]
isogroup07101	NA
isogroup07159	putative stathmin-like 3 [Taeniopygia guttata]
isogroup07261	NA
isogroup07275	NA
isogroup07353	PREDICTED: GRIP and coiled-coil domain containing 2 [Taeniopygia guttata]
isogroup07379	PREDICTED: similar to protocadherin 9 [Sus scrofa]
isogroup07381	PREDICTED: SATB homeobox 1 [Taeniopygia guttata]
isogroup07784	NA
isogroup07848	NA
isogroup07871	PREDICTED: TNF receptor-associated factor 3 isoform 1 [Taeniopygia guttata]
isogroup08524	NA
isogroup08753	NA
isogroup09194	NA
isogroup09293	POL-like [Gallus gallus]
isogroup09388	PREDICTED: TraB domain containing [Taeniopygia guttata]
isogroup09413	unknown [Gallus gallus]
isogroup10025	PREDICTED: similar to family with sequence similarity 102, member A [Taeniopygia guttata]

Isogroup	Annotation from nr Database
isogroup10114	PREDICTED: cytoglobin [Taeniopygia guttata]
isogroup10165	NA
isogroup10211	NA
isogroup10365	PREDICTED: chromosome 22 open reading frame 13 [Taeniopygia guttata]
isogroup10565	PREDICTED: myeloid/lymphoid or mixed-lineage leukemia (trithorax homolog, Drosophila) [Taeniopygia guttata]
isogroup10638	NA
isogroup10647	NA
isogroup10703	NA
isogroup10773	PREDICTED: hypothetical protein [Taeniopygia guttata]
isogroup10827	PREDICTED: similar to formin 2 [Taeniopygia guttata]
isogroup10880	PREDICTED: ankyrin repeat domain 12 [Taeniopygia guttata]
isogroup10955	NA
isogroup11785	NA
isogroup11822	NA
isogroup12018	NA
isogroup12084	PREDICTED: basic beta 1 syntrophin [Taeniopygia guttata]
isogroup12109	NA
isogroup12138	PREDICTED: similar to KIAA0833 protein [Taeniopygia guttata]
isogroup12192	NA
isogroup12421	NA
isogroup12451	PREDICTED: RALBP1 associated Eps domain containing 2 [Taeniopygia guttata]
isogroup12666	PREDICTED: similar to chorea-acanthocytosis [Taeniopygia guttata]
isogroup12838	NA
isogroup12966	PREDICTED: similar to SLIT and NTRK-like family, member 4 [Taeniopygia guttata]
isogroup12980	NA
isogroup13155	PREDICTED: regulator of G-protein signalling 7 [Taeniopygia guttata]
isogroup13297	NA
isogroup13324	NA
isogroup13483	NA
isogroup13518	NA
isogroup13725	PREDICTED: centrosomal protein 290kDa [Taeniopygia guttata]
isogroup13767	NA
isogroup13774	PREDICTED: chromosome 7 open reading frame 16 [Taeniopygia guttata]
isogroup13900	AF362753_1 cer-d4 [Gallus gallus]
isogroup13948	PREDICTED: ryanodine receptor 2 (cardiac) [Taeniopygia guttata]

Isogroup	Annotation from nr Database
isogroup14159	PREDICTED: ubiquitin specific peptidase 13 (isopeptidase T-3) [Taeniopygia guttata]
isogroup14299	NA
isogroup14377	NA
isogroup14692	PREDICTED: similar to ribosomal protein L7-like 1 [Taeniopygia guttata]
isogroup15075	NA
isogroup15096	chromodomain-helicase-DNA-binding protein 7 [Gallus gallus]
isogroup15222	NA
isogroup15235	NA
isogroup15269	PREDICTED: similar to centrosomal protein 2 [Gallus gallus]
isogroup15454	NA
isogroup15468	PREDICTED: protocadherin 9 [Taeniopygia guttata]
isogroup15486	interferon-related developmental regulator 1 [Gallus gallus]
isogroup15693	NA
isogroup15730	NA
isogroup15768	PREDICTED: similar to collagen, type XII, alpha 1 [Taeniopygia guttata]
isogroup16272	NA
isogroup16714	PREDICTED: RAS p21 protein activator 2 [Taeniopygia guttata]
isogroup16821	PREDICTED: similar to myosin 10 [Taeniopygia guttata]
isogroup16893	NA
isogroup16963	PREDICTED: similar to mutS homolog 4 (E. coli), partial [Taeniopygia guttata]
isogroup17432	PREDICTED: similar to Nesprin-1 (Nuclear envelope spectrin repeat protein 1) (Synaptic nuclear envelope protein 1) (Syne-1) (Myocyte nuclear envelope protein 1) (Myne-1) (Enaptin) [Gallus gallus]
isogroup17447	chromodomain-helicase-DNA-binding protein 7 [Gallus gallus]
isogroup17481	NA
isogroup17492	NA
isogroup17626	NA
isogroup17828	NA
isogroup17831	NA
isogroup18038	NA
isogroup18155	NA
isogroup18197	NA
isogroup18244	NA
isogroup18339	NA
isogroup18352	NA
isogroup18608	PREDICTED: similar to copine III isoform 2 [Taeniopygia guttata]

Isogroup	Annotation from nr Database
isogroup18724	PREDICTED: splicing factor, arginine/serine-rich 18 [Taeniopygia guttata]
isogroup18942	NA
isogroup18953	PREDICTED: similar to leucine rich repeat containing 8 family, member B [Taeniopygia guttata]
isogroup19062	PREDICTED: similar to collagen, type XII, alpha 1 [Taeniopygia guttata]
isogroup19160	PREDICTED: solute carrier family 12 (sodium/potassium/chloride transporters), member 2 [Taeniopygia guttata]
isogroup19477	PREDICTED: hypothetical protein [Taeniopygia guttata]
isogroup19617	NA
isogroup19912	PREDICTED: similar to solute carrier family 4, sodium bicarbonate transporter-like, member 10 [Ornithorhynchus anatinus]
isogroup20449	PREDICTED: similar to spastic ataxia of Charlevoix-Saguenay (sacsin) [Gallus gallus]
isogroup20476	NA
isogroup20497	NA
isogroup20549	NA
isogroup20701	chromodomain-helicase-DNA-binding protein 7 [Gallus gallus]
isogroup20808	NA
isogroup20821	NA
isogroup20959	PREDICTED: hypothetical protein [Taeniopygia guttata]
isogroup21032	NA
isogroup21056	NA
isogroup21069	NA
isogroup21083	SPHK1 interactor, AKAP domain containing [Taeniopygia guttata]
isogroup21379	NA
isogroup21987	PREDICTED: hypothetical protein [Gallus gallus]
isogroup22031	NA
isogroup22221	PREDICTED: similar to RAS protein activator like 2 [Gallus gallus]
isogroup22484	AF355752_1 reverse transcriptase [Chelonia mydas]
isogroup22534	NA
isogroup22581	NA
isogroup22638	PREDICTED: transient receptor potential cation channel, subfamily M, member 8 [Taeniopygia guttata]
isogroup22721	ORF2 [Platemys spixii]
contig90074	NA
contig90116	NA
contig90192	NA

Isogroup	Annotation from nr Database
contig90199	NA
contig90201	NA

Supplementary Table 10. Genes expressed lower in T-treated than control females in the medial amygdala.

Isogroup	Annotation from nr Database
isogroup00584	PREDICTED: phosphotriesterase related [Taeniopygia guttata]
isogroup00650	NA
isogroup01101	PREDICTED: mitochondrial ribosomal protein L18 [Taeniopygia guttata]
isogroup02339	PREDICTED: similar to opioid receptor, kappa 1 [Taeniopygia guttata]
isogroup02915	PREDICTED: similar to heat shock protein 70kDa [Taeniopygia guttata]
isogroup03014	PREDICTED: sperm associated antigen 16 [Taeniopygia guttata]
isogroup03227	NA
isogroup03438	PREDICTED: hypothetical protein [Gallus gallus]
isogroup03755	putative neurotensin variant 1 [Taeniopygia guttata]
isogroup04378	NA
isogroup04509	PREDICTED: similar to Adenylate kinase isoenzyme 5, partial [Taeniopygia guttata]
isogroup04887	PREDICTED: enoyl Coenzyme A hydratase domain containing 2 [Taeniopygia guttata]
isogroup04912	NA
isogroup05026	PREDICTED: hypothetical protein [Gallus gallus]
isogroup05078	NA
isogroup05100	PREDICTED: monoamine oxidase A [Taeniopygia guttata]
isogroup05198	PREDICTED: similar to polymerase [Taeniopygia guttata]
isogroup05737	putative ribosomal protein S19 [Taeniopygia guttata]
isogroup06188	NA
isogroup06191	PREDICTED: similar to Discoidin, CUB and LCCL domain-containing protein 1 [Taeniopygia guttata]
isogroup06326	tumor necrosis factor, alpha-induced protein 6 [Gallus gallus]
isogroup06451	PREDICTED: parvalbumin [Taeniopygia guttata]
isogroup06491	NA
isogroup06530	NA
isogroup06945	PREDICTED: similar to Tetratricopeptide repeat domain 39A [Taeniopygia guttata]
isogroup07236	NA
isogroup07264	NA
isogroup07330	NA
isogroup07371	NA
isogroup07544	PREDICTED: hypothetical protein [Taeniopygia guttata]
isogroup07899	putative Schwann cell-specific EGF-like repeat autocrine factor [Taeniopygia guttata]
isogroup07917	NA
isogroup07997	NA

Isogroup	Annotation from nr Database
isogroup08029	NA
isogroup08034	DNA-dependent protein kinase catalytic subunit [Gallus gallus]
isogroup08059	NA
isogroup08135	PREDICTED: WD repeat domain 5 [Taeniopygia guttata]
isogroup08243	NA
isogroup08283	NA
isogroup08341	NA
isogroup08480	NA
isogroup08606	NA
isogroup08732	NA
isogroup08788	NA
isogroup09028	PREDICTED: similar to autoimmune infertility-related protein [Taeniopygia guttata]
isogroup09044	NA
isogroup09053	NA
isogroup09539	NA
isogroup09648	NA
isogroup09791	PREDICTED: FK506 binding protein 14, 22 kDa [Taeniopygia guttata]
isogroup09831	NA
isogroup09947	NA
isogroup09973	NA
isogroup09978	NA
isogroup10032	NA
isogroup10208	NA
isogroup10233	PREDICTED: hypothetical protein [Taeniopygia guttata]
isogroup10242	NA
isogroup10244	NA
isogroup10401	NA
isogroup10403	NA
isogroup10681	actin related protein 2/3 complex subunit 5 [Gallus gallus]
isogroup10848	PREDICTED: suppressor of G2 allele of SKP1 [Taeniopygia guttata]
isogroup10861	PREDICTED: hypothetical protein [Taeniopygia guttata]
isogroup10914	NA
isogroup11062	PREDICTED: hypothetical protein [Taeniopygia guttata]
isogroup11139	PREDICTED: solute carrier family 47, member 2 [Taeniopygia guttata]
isogroup11319	NA
isogroup11438	NA

Isogroup	Annotation from nr Database
isogroup11507	NA
isogroup12010	PREDICTED: reprimo-like [Taeniopygia guttata]
isogroup12351	NA
isogroup12597	NA
isogroup12757	NA
isogroup12920	PREDICTED: similar to alphaT-catenin [Gallus gallus]
isogroup13184	NA
isogroup13307	PREDICTED: monoamine oxidase A [Taeniopygia guttata]
isogroup13347	NA
isogroup13403	PREDICTED: putative ribosomal protein S11 [Taeniopygia guttata]
isogroup13509	NA
isogroup13715	NA
isogroup13716	PREDICTED: similar to tetraspanin 6 [Gallus gallus]
isogroup14050	PREDICTED: putative preproenkephalin 1 [Taeniopygia guttata]
isogroup14211	PREDICTED: hypothetical protein [Taeniopygia guttata]
isogroup14300	NA
isogroup14407	NA
isogroup14447	putative RIKEN cDNA 2900055D14 [Taeniopygia guttata]
isogroup14539	NA
isogroup14565	putative actin related protein 2/3 complex subunit 3 [Taeniopygia guttata]
isogroup14780	NA
isogroup14837	PREDICTED: putative mitochondrial ribosomal protein S14 [Taeniopygia guttata]
isogroup14908	NA
isogroup14947	NA
isogroup14976	NA
isogroup15108	NA
isogroup15127	hypothetical protein PANDA_012261 [Ailuropoda melanoleuca]
isogroup15141	PREDICTED: similar to Gpr7 [Taeniopygia guttata]
isogroup15208	NA
isogroup15405	PREDICTED: similar to Sucrase-isomaltase, intestinal [Gallus gallus]
isogroup15617	ribosomal protein S13 [synthetic construct]
isogroup15823	PREDICTED: galanin receptor 3 [Taeniopygia guttata]
isogroup15923	NA
isogroup16058	NA
isogroup16245	NA
isogroup16415	NA

Isogroup	Annotation from nr Database
isogroup16468	PREDICTED: similar to heparin cofactor II [Gallus gallus]
isogroup16521	NA
isogroup16558	PREDICTED: similar to DTW domain containing 1 [Taeniopygia guttata]
isogroup16574	PREDICTED: protein tyrosine phosphatase, receptor type, M [Taeniopygia guttata]
isogroup16576	PREDICTED: hypothetical protein isoform 2 [Pan troglodytes]
isogroup16611	NA
isogroup16703	NA
isogroup16804	PREDICTED: similar to insulinoma protein (rig) [Monodelphis domestica]
isogroup16824	PREDICTED: LIM homeobox 8 [Taeniopygia guttata]
isogroup17012	mCG49427 [Mus musculus]
isogroup17169	NA
isogroup17178	NA
isogroup17181	NA
isogroup17559	PREDICTED: similar to leucine rich repeat transmembrane neuronal 1 [Taeniopygia guttata]
isogroup17664	PREDICTED: sclerostin domain containing 1 [Taeniopygia guttata]
isogroup17717	NA
isogroup17788	NA
isogroup17968	NA
isogroup18039	PREDICTED: similar to Leucine rich repeat containing 56 [Monodelphis domestica]
isogroup18049	tumor necrosis factor receptor superfamily, member 21 [Zonotrichia albicollis]
isogroup18184	PREDICTED: centrin, EF-hand protein, 1 [Taeniopygia guttata]
isogroup18286	NA
isogroup18439	NA
isogroup18768	NA
isogroup18823	NA
isogroup19158	PREDICTED: interleukin 18 (interferon-gamma-inducing factor) [Taeniopygia guttata]
isogroup19195	PREDICTED: similar to rCG28794 [Taeniopygia guttata]
isogroup19269	NA
isogroup19455	NA
isogroup19720	NA
isogroup19752	PREDICTED: hypothetical protein [Taeniopygia guttata]
isogroup19929	NA
isogroup19932	PREDICTED: hairy and enhancer of split 5 isoform 1 [Taeniopygia guttata]
isogroup20545	NA

Isogroup	Annotation from nr Database
isogroup20559	PREDICTED: RPA interacting protein [Taeniopygia guttata]
isogroup20567	PREDICTED: monooxygenase, DBH-like 1 [Taeniopygia guttata]
isogroup20624	NA
isogroup20723	PREDICTED: golgi autoantigen, golgin subfamily a, 7 [Taeniopygia guttata]
isogroup20813	signal peptidase complex subunit 1 homolog [Gallus gallus]
isogroup21215	NA
isogroup21241	PREDICTED: similar to ATPase, H+ transporting, lysosomal V0 subunit a isoform 2 [Canis familiaris]
isogroup21277	PREDICTED: interleukin 16 [Taeniopygia guttata]
isogroup21319	NA
isogroup21632	NA
isogroup21653	NA
isogroup21676	NA
isogroup21706	NA
isogroup21960	NA
isogroup22057	NA
isogroup22080	NA
isogroup22180	NA
isogroup22589	NA
isogroup22713	NA
isogroup22723	PREDICTED: hypothetical protein [Gallus gallus]
contig90154	NA

Supplementary Table 11. Gene Ontology terms over-represented among genes significantly differentially expressed between T-treated and control females in the medial amygdala.

GO ID	GO Description
GO:0001505	regulation of neurotransmitter levels
GO:0001944	vasculature development
GO:0003013	circulatory system process
GO:0008344	adult locomotory behavior
GO:0008406	gonad development
GO:0021675	nerve development
GO:0030252	growth hormone secretion
GO:0030326	embryonic limb morphogenesis
GO:0031109	microtubule polymerization or depolymerization
GO:0035264	multicellular organism growth
GO:0042471	ear morphogenesis
GO:0043010	camera-type eye development
GO:0046545	development of primary female sexual characteristics
GO:0005198	structural molecule activity
GO:0008528	G-protein coupled peptide receptor activity
GO:0005840	ribosome
GO:0015935	small ribosomal subunit
GO:0035097	histone methyltransferase complex

Supplementary Table 12. Genes expressed higher in T-treated than control females in hypothalamus.

Isogroup	Annotation from nr Database
isogroup01688	PREDICTED: similar to MBP-2 (MHC Binding Protein-2) [Taeniopygia guttata]
isogroup01966	NA
isogroup02363	cytochrome P450, family 19, subfamily A, polypeptide 1 [Taeniopygia guttata]
isogroup02560	unnamed protein product [Homo sapiens]
isogroup02830	NA
isogroup03358	PREDICTED: similar to EEF2K protein [Taeniopygia guttata]
isogroup04503	NA
isogroup04575	PREDICTED: hypothetical protein [Taeniopygia guttata]
isogroup04715	PREDICTED: similar to CDC42 binding protein kinase beta (DMPK-like) [Gallus gallus]
isogroup04886	NA
isogroup05219	PREDICTED: similar to eukaryotic translation initiation factor 4 gamma, 3 [Gallus gallus]
isogroup05441	NA
isogroup05946	NA
isogroup06072	PREDICTED: similar to plasma membrane calcium ATPase 2 isoform 5 [Gallus gallus]
isogroup06893	PREDICTED: hypothetical protein [Taeniopygia guttata]
isogroup07713	NA
isogroup08448	PREDICTED: IQ motif containing E [Taeniopygia guttata]
isogroup08467	NA
isogroup08624	PREDICTED: transmembrane protein 55A [Taeniopygia guttata]
isogroup08921	NA
isogroup09761	PREDICTED: similar to prominin-like 1 [Taeniopygia guttata]
isogroup12451	PREDICTED: RALBP1 associated Eps domain containing 2 [Taeniopygia guttata]
isogroup14159	PREDICTED: ubiquitin specific peptidase 13 (isopeptidase T-3) [Taeniopygia guttata]
isogroup14232	PREDICTED: F-box only protein 32 [Taeniopygia guttata]
isogroup14234	PREDICTED: ryanodine receptor 2 (cardiac) [Taeniopygia guttata]
isogroup14253	NA
isogroup14354	PREDICTED: similar to MBP-2 (MHC Binding Protein-2) [Taeniopygia guttata]
isogroup14692	PREDICTED: similar to ribosomal protein L7-like 1 [Taeniopygia guttata]
isogroup14839	NA
isogroup15212	NA
isogroup15454	NA

Isogroup	Annotation from nr Database
isogroup15707	PREDICTED: human immunodeficiency virus type I enhancer binding protein 1 [Taeniopygia guttata]
isogroup15736	PREDICTED: lipoprotein lipase [Taeniopygia guttata]
isogroup15794	PREDICTED: ArfGAP with GTPase domain, ankyrin repeat and PH domain 1 [Taeniopygia guttata]
isogroup15864	cannabinoid receptor 1 (brain) [Taeniopygia guttata]
isogroup16272	NA
isogroup17074	PREDICTED: ryanodine receptor 2 (cardiac) [Taeniopygia guttata]
isogroup17170	calcineurin A alpha [Gallus gallus]
isogroup17550	PREDICTED: ryanodine receptor 2 (cardiac) [Taeniopygia guttata]
isogroup18416	NA
isogroup18473	PREDICTED: similar to MGC115225 protein [Taeniopygia guttata]
isogroup19083	PREDICTED: latrophilin 3 [Equus caballus]
isogroup19864	NA
isogroup20240	NA
isogroup20578	NA
isogroup20987	transmembrane channel-like 7 [Gallus gallus]
isogroup21068	PREDICTED: similar to SETDB2 protein [Taeniopygia guttata]
isogroup21131	NA
isogroup22201	PREDICTED: similar to CXXC finger 6 [Taeniopygia guttata]

Supplementary Table 13. Genes expressed lower in T-treated than control females in hypothalamus.

Isogroup	Annotation from nr Database
isogroup00934	PREDICTED: ADP-ribosylation factor binding protein 3 [Taeniopygia guttata]
isogroup01698	PREDICTED: tumor necrosis factor receptor superfamily, member 11b (osteoprotegerin) [Taeniopygia guttata]
isogroup02599	NA
isogroup05794	NA
isogroup06596	PREDICTED: similar to HSPC287 [Taeniopygia guttata]
isogroup07298	PREDICTED: family with sequence similarity 108, member B1 [Taeniopygia guttata]
isogroup07371	NA
isogroup09237	NA
isogroup09253	signal sequence receptor, beta, isoform CRA_a [Mus musculus]
isogroup09356	PREDICTED: hypothetical protein LOC100190396 [Taeniopygia guttata]
isogroup11399	PREDICTED: hypothetical protein [Gallus gallus]
isogroup11507	NA
isogroup11590	NA
isogroup11764	NA
isogroup11851	NA
isogroup12176	NA
isogroup12920	PREDICTED: similar to alphaT-catenin [Gallus gallus]
isogroup13234	PREDICTED: hypothetical protein [Taeniopygia guttata]
isogroup14417	NA
isogroup15113	NA
isogroup17568	PREDICTED: WNT inhibitory factor 1 [Taeniopygia guttata]
isogroup17584	NA
isogroup17717	NA
isogroup18342	placenta-specific 8 [Mus musculus]
isogroup19158	PREDICTED: interleukin 18 (interferon-gamma-inducing factor) [Taeniopygia guttata]
isogroup19446	NA
isogroup19455	NA
isogroup19660	PREDICTED: similar to MTERF domain containing 3 [Taeniopygia guttata]
isogroup20422	neuropeptide Y precursor [Gallus gallus]
isogroup22713	NA

Supplementary Table 14. Gene Ontology terms over-represented among genes significantly differentially expressed between T-treated and control females in the hypothalamus.

GO ID	GO Description
GO:0006941	striated muscle contraction
GO:0007186	G-protein coupled receptor signaling pathway
GO:0008624	induction of apoptosis by extracellular signals
GO:0009593	detection of chemical stimulus
GO:0010038	response to metal ion
GO:0010880	regulation of release of sequestered calcium ion into cytosol by sarcoplasmic reticulum
GO:0014074	response to purine-containing compound
GO:0031667	response to nutrient levels
GO:0070838	divalent metal ion transport
GO:0005218	intracellular ligand-gated calcium channel activity
GO:0046872	metal ion binding
GO:0051018	protein kinase A binding
GO:0005789	endoplasmic reticulum membrane
GO:0031674	I band
GO:0034702	ion channel complex

Supplementary Table 15. Genes expressed higher in T-treated than control females in both the medial amygdala and hypothalamus.

**Isogroup**	**Annotation from nr Database**
isogroup04503	NA
isogroup04886	NA
isogroup05219	PREDICTED: similar to eukaryotic translation initiation factor 4 gamma, 3 [Gallus gallus]
isogroup05946	NA
isogroup12451	PREDICTED: RALBP1 associated Eps domain containing 2 [Taeniopygia guttata]
isogroup14159	PREDICTED: ubiquitin specific peptidase 13 (isopeptidase T-3) [Taeniopygia guttata]
isogroup14692	PREDICTED: similar to ribosomal protein L7-like 1 [Taeniopygia guttata]
isogroup15454	NA
isogroup16272	NA

Supplementary Table 16. Genes expressed lower in T-treated than control females in both the medial amygdala and hypothalamus.

Isogroup	Annotation from nr Database
isogroup07371	NA
isogroup11507	NA
isogroup12920	PREDICTED: similar to alphaT-catenin [Gallus gallus]
isogroup17717	NA
isogroup19158	PREDICTED: interleukin 18 (interferon-gamma-inducing factor) [Taeniopygia guttata]
isogroup19455	NA
isogroup22713	NA

Supplementary Table 17. Genes expressed higher in T-treated than control males in the medial amygdala.

Isogroup	Annotation from nr Database
isogroup03488	putative MGC89063 protein variant 2 [Taeniopygia guttata]
isogroup05526	PREDICTED: similar to GekBS018P [Taeniopygia guttata]
isogroup06043	PREDICTED: carboxypeptidase A2 (pancreatic) [Taeniopygia guttata]
isogroup07410	PREDICTED: testin [Taeniopygia guttata]
isogroup08239	PREDICTED: similar to inositol-1,4,5-trisphosphate-3-kinase, partial [Taeniopygia guttata]
isogroup09891	R envelope protein [Pongo pygmaeus]
isogroup10000	NA
isogroup14755	PREDICTED: similar to GABA-A receptor alpha-3 subunit [Monodelphis domestica]
isogroup17230	PREDICTED: similar to solute carrier family 30 (zinc transporter), member 10 [Taeniopygia guttata]
isogroup18791	ORF2 [Platemys spixii]
isogroup18901	PREDICTED: similar to complement C4 [Taeniopygia guttata]
isogroup19308	PREDICTED: solute carrier family 18 (vesicular monoamine), member 2 [Taeniopygia guttata]
isogroup19873	PREDICTED: similar to sulfonylurea receptor isoform 1 [Gallus gallus]
isogroup21866	NA
contig90180	NA

Supplementary Table 18. Genes expressed lower in T-treated than control males in the medial amygdala.

Isogroup	Annotation from nr Database
isogroup03275	PREDICTED: similar to solute carrier family 7, (neutral amino acid transporter, y+ system) member 10 [Taeniopygia guttata]
isogroup04720	PREDICTED: non-imprinted in Prader-Willi/Angelman syndrome 1 [Taeniopygia guttata]
isogroup05470	NA
isogroup05496	PREDICTED: osteoglycin [Taeniopygia guttata]
isogroup06584	PREDICTED: similar to transcription factor AP2-alpha isoform 2 [Ornithorhynchus anatinus]
isogroup07415	NA
isogroup09588	NA
isogroup10647	NA
isogroup14971	PREDICTED: similar to chondroadherin [Taeniopygia guttata]
isogroup16303	PREDICTED: similar to Hepatocyte nuclear factor 6 (HNF-6) (One cut domain family member 1) isoform 1 [Canis familiaris]
isogroup16448	NA
isogroup16526	PREDICTED: similar to potassium inwardly-rectifying channel J13 [Taeniopygia guttata]
isogroup17579	NA
isogroup18736	NA
isogroup19239	NA
isogroup20010	Hox-8 [Gallus gallus]
isogroup20096	NA
isogroup20312	PREDICTED: similar to retinoic acid receptor responder (tazarotene induced) 1 [Taeniopygia guttata]
isogroup20809	PREDICTED: solute carrier organic anion transporter family, member 1C1 [Taeniopygia guttata]
isogroup21077	NA
isogroup22638	PREDICTED: transient receptor potential cation channel, subfamily M, member 8 [Taeniopygia guttata]

Supplementary Table 19. Genes expressed higher in T-treated than control males in hypothalamus.

Isogroup	Annotation from nr Database
isogroup00789	NA
isogroup01128	PREDICTED: similar to KIAA1882 protein [Gallus gallus]
isogroup02136	PREDICTED: similar to carotene-9,10-monooxygenase [Taeniopygia guttata]
isogroup02234	PREDICTED: similar to KIAA1882 protein [Gallus gallus]
isogroup02363	cytochrome P450, family 19, subfamily A, polypeptide 1 [Taeniopygia guttata]
isogroup03488	putative MGC89063 protein variant 2 [Taeniopygia guttata]
isogroup04057	NA
isogroup04099	NA
isogroup05819	NA
isogroup06043	PREDICTED: carboxypeptidase A2 (pancreatic) [Taeniopygia guttata]
isogroup06326	tumor necrosis factor, alpha-induced protein 6 [Gallus gallus]
isogroup06604	PREDICTED: glycerol-3-phosphate acyltransferase, mitochondrial [Taeniopygia guttata]
isogroup06970	DNA-dependent protein kinase catalytic subunit [Gallus gallus]
isogroup07064	NA
isogroup07410	PREDICTED: testin [Taeniopygia guttata]
isogroup07558	PREDICTED: similar to Transmembrane anchor protein 1 [Gallus gallus]
isogroup07950	MC4R [Anser anser]
isogroup08261	NA
isogroup09891	R envelope protein [Pongo pygmaeus]
isogroup10152	NA
isogroup11817	NA
isogroup13009	NA
isogroup14243	PREDICTED: hypothetical protein [Gallus gallus]
isogroup14777	NA
isogroup15188	PREDICTED: similar to panopsin [Gallus gallus]
isogroup16513	PREDICTED: phosphoglucomutase 1 isoform 1 [Taeniopygia guttata]
isogroup16847	NA
isogroup17168	PREDICTED: paraoxonase 2 isoform 1 [Taeniopygia guttata]
isogroup18351	PREDICTED: glycerol-3-phosphate acyltransferase, mitochondrial [Taeniopygia guttata]
isogroup19711	PREDICTED: cytospin A [Taeniopygia guttata]
isogroup19740	NA
isogroup19850	NA
isogroup21172	NA

Isogroup	Annotation from nr Database
isogroup21278	PREDICTED: similar to gliomedin [Taeniopygia guttata]
contig90068	NA

Supplementary Table 20. Genes expressed lower in T-treated than control males in hypothalamus.

Isogroup	Annotation from nr Database
isogroup01140	NA
isogroup01574	PREDICTED: splicing factor, arginine/serine-rich 5 [Taeniopygia guttata]
isogroup02347	NA
isogroup02469	putative protein kinase C inhibitor/ASWZ [Taeniopygia guttata]
isogroup03391	NA
isogroup03738	NA
isogroup04207	NA
isogroup04627	PREDICTED: regulating synaptic membrane exocytosis 2 isoform 2 [Taeniopygia guttata]
isogroup05307	NA
isogroup05336	PREDICTED: sulfatase modifying factor 2 isoform 1 [Taeniopygia guttata]
isogroup05470	NA
isogroup06195	PREDICTED: protein phosphatase 4, regulatory subunit 1 [Taeniopygia guttata]
isogroup07467	PREDICTED: interferon regulatory factor 1 [Taeniopygia guttata]
isogroup09194	NA
isogroup09735	dicer 1, ribonuclease type III [Taeniopygia guttata]
isogroup09959	NA
isogroup10184	NA
isogroup10647	NA
isogroup11316	PREDICTED: similar to LIM-homeobox 9 isoform 2 [Monodelphis domestica]
isogroup12602	NA
isogroup12750	NA
isogroup12816	NA
isogroup13274	NA
isogroup13380	NA
isogroup13641	PREDICTED: calbindin 1, 28kDa [Taeniopygia guttata]
isogroup14823	PREDICTED: similar to chorea-acanthocytosis [Taeniopygia guttata]
isogroup14931	PREDICTED: hypothetical protein [Gallus gallus]
isogroup15235	NA
isogroup15384	methylcrotonoyl-Coenzyme A carboxylase 2 (beta) [Xenopus laevis]
isogroup16120	PREDICTED: similar to natural killer tumor recognition protein [Gallus gallus]
isogroup16428	PREDICTED: fibroblast growth factor 14 isoform 1 [Taeniopygia guttata]
isogroup16685	NA
isogroup16695	PREDICTED: ubiquitin specific peptidase like 1 [Taeniopygia guttata]
isogroup17199	POL-like [Gallus gallus]

Isogroup	Annotation from nr Database
isogroup18023	NA
isogroup18086	NA
isogroup18469	growth hormone releasing hormone [Gallus gallus]
isogroup18599	NA
isogroup18689	NA
isogroup18880	NA
isogroup19057	PREDICTED: similar to protein tyrosine phosphatase type 1, partial [Ornithorhynchus anatinus]
isogroup19524	NA
isogroup20096	NA
isogroup20696	PREDICTED: unc-13 homolog C (C. elegans) [Taeniopygia guttata]
isogroup21210	NA
isogroup21289	NA
isogroup21497	NA
isogroup21580	NA
isogroup22021	NA
isogroup22327	NA
isogroup22534	NA
isogroup22581	NA
isogroup22638	PREDICTED: transient receptor potential cation channel, subfamily M, member 8 [Taeniopygia guttata]
contig90097	NA

Supplementary Table 21. Genes expressed higher in T-treated than control males in both the medial amygdala and hypothalamus.

Isogroup	Annotation from nr Database
isogroup03488	putative MGC89063 protein variant 2 [Taeniopygia guttata]
isogroup06043	PREDICTED: carboxypeptidase A2 (pancreatic) [Taeniopygia guttata]
isogroup07410	PREDICTED: testin [Taeniopygia guttata]
isogroup09891	R envelope protein [Pongo pygmaeus]

Supplementary Table 22. Genes expressed lower in T-treated than control males in both the medial amygdala and hypothalamus.

Isogroup	Annotation from nr Database
isogroup05470	NA
isogroup10647	NA
isogroup20096	NA
isogroup22638	PREDICTED: transient receptor potential cation channel, subfamily M, member 8 [Taeniopygia guttata]
